# Epidemiology of chronic rhinosinusitis, selected risk factors, comorbidities, and economic burden

**DOI:** 10.3205/cto000126

**Published:** 2015-12-22

**Authors:** Achim Beule

**Affiliations:** 1 ENT Department, University of Greifswald, Germany

**Keywords:** epidemiology, prevalence, chronic rhinosinusitis, risk factor, asthma, endoscopic sinus surgery, economy, smoking, immunosuppression, occupational hazard

## Abstract

Chronic rhinosinusitis (CRS) is a relevant and prevalent medical condition in Germany, Europe and the world. If analysed in detail, the prevalence of CRS shows regional and temporary variety. In this review, currently available data regarding the prevalence of CRS is therefore sorted by country and/or region, time point of data collection and the CRS-definition employed. Risk factors like smoking and gastroesophageal reflux are discussed regarding their influence on CRS prevalence. Moreover, comorbidities of CRS, like asthma, conditions of the cardiovascular system and depression are listed and their influence on CRS is discussed. Furthermore, data on CRS prevalence in special cohorts, like immunocompromised patients, are presented. To estimate the economic burden of CRS, current data e.g. from Germany and the USA are included in this review.

## 1 Epidemiologic data on chronic rhinosinusitis (CRS)

### 1.1 Preface

The basis of the following review article are Medline and PubMed listed publications as well as data published on the internet pages of the National Institute of Health. Because of the fact that epidemiologic datasets cannot be found on centrally organized sources, this review article does not claim to be exhaustive or complete.

### 1.2 Introduction

Reliable data on the epidemiology of chronic rhinosinusitis (CRS) and especially of their subtypes are still rare.

That is why the European Rhinologic Society made great efforts to improve this situation. Based on the GA^2^LEN study (Global Allergy and Asthma European Network; http://www.ga2len.net/) and preparatory and accompanying consensus publications, significant progress could be achieved. Numerous aspects, among others also the aspect of epidemiology, were assembled in the EPOS paper of 2012 [1] and will be evaluated here from a German point of view and described in an actualized way.

Unfortunately, the results cannot be communicated in the descriptive and easily comparable manner that would be desirable. This is especially due to the insufficient data situation, the varying methods of investigation, and the differences in the social and economic circumstances of the single countries. Further, regional differences must be mentioned. In the following, evaluations are classified according to their origin: Germany and Europe, USA and Canada, Asiatic countries, and the rest of world if possible. This gradual approach will take into consideration the regional differences with regard to pathophysiology of the chronic rhinosinusitis on the one hand and the nutritional habits, the social and genetic differences of the population on the other hand.

Despite all limitations, the following data on the epidemiology of chronic rhinosinusitis of the German ENT practice will allow a better view on all described diseases and serve as a basis for individual investigations.

CRS is a general term for a pathophysiologically heterogeneous group of diseases. Up to now, this fact has only been considered with regard to the collection of epidemiologic data for the group of CRS with nasal polyps (CRSwNP). However, in order to be able to give statements on subgroups of CRS, relative data, e.g. on the incidence of different types of fungal sinusitis, have been included in the evaluation.

### 1.3 Explanation of epidemiologic terms

In the following, frequently used terms of the field of epidemiology will be presented in order to allow a better understanding of the data, e.g. the odds ratio in the context of CRS. This list (Table 1 (Tab. 1)) is deliberately limited to the present review.

**Figure 1:**
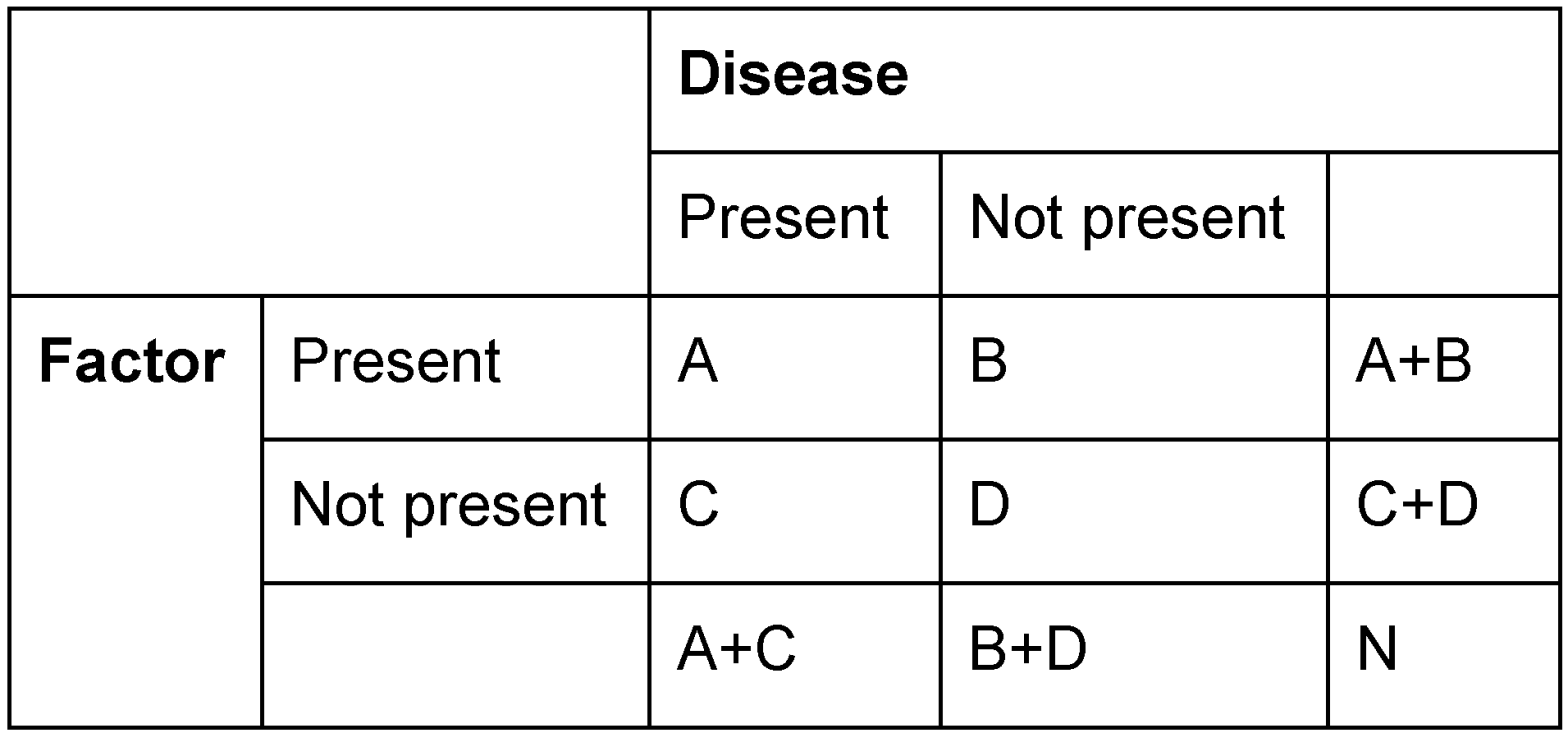
Fourfold table and formulas for calculation of epidemiological parameters This figure shows a fourfold table with classification into presence/absence of a disease and a factor (e.g. a risk factor). In synopsis of the formulas of Tab. 2 the epidemiologic ratio can be calculated.

The used terms are mathematically based on a fourfold table (Figure 1 (Fig. 1)) that does not only show the calculation base but also helps to understand the variety of the terms.

## 2 Epidemiology of chronic rhinosinusitis and their subtypes

### 2.1 Prevalence of sinusitis

In the scientific literature there are often hints to data of the Center of Disease Control (CDC) mentioned as source for the prevalence of chronic sinusitis. The CDC with its department of the National Center for Health Statistics (NCHS) carries out population-based investigations in the USA since 1956 regarding the incidence of diseases and hence can provide the oldest series of population-based data for example on the prevalence of sinusitis. For 1990–1992, chronic sinusitis – based on the encoding of the ICD codes 473 for symptoms that persist for more than 8 weeks – is considered to be the second most common disease in the USA [2]. Comparable data from Germany and/or Europe are not present. A current evaluation from South America reports 16.55% for rhinosinusitis in the context of a pilot study (CI: 14.18–19.23) [3] and thus confirms the scale Reporter in the USA (Table 2 (Tab. 2)).

Table 3 (Tab. 3) lists the publications of the CDC/NIHS that are based on a survey of adults. Those interviews only contained the question if the people suffered from “sinusitis”. 

It seems astonishing, that the extensive American investigations do not consider the difference between acute and chronic sinusitis. Despite methodical changes of the CDC/NIHS (reduction of the sample size by 13% since 2006 and the evaluation of age-related data since 2002) and the systematic underestimation of the incidence because of exclusions of participants who did not reply, there seems to be a slightly decreasing, but relevant group of people in the USA suffering from “sinusitis”. Females seem to be more frequently affected than males. Based on the way of investigation, influences such as an easier access to sinusitis treatment for females or an increased health awareness of females as well as a different gender preference of various sinusitis types cannot be delineated.

For the USA, another evaluation came to the conclusion of a prevalence of recurrent acute rhinosinusitis of 0.035% [4]. According to those data, the prevalence of chronic rhinosinusitis is expected to currently amount to ≤12% in the USA because singularly occurring types of sinusitis are also considered.

Until 2014, there was no general health insurance in the USA, and so 15% of the American population had no access to services of the insurance system. Because of this fact, an undersupply in about 1/6 of the population can be expected. Another 8% of the population have a “direct insurance” (comparable to the private health insurance system in Germany) so that the individual economic situation may have an impact on the use of services of the health system. Based on the economic crisis that has also reached the USA, this could also be an explanation for the reduced number of diagnoses of sinusitis during the last years. The degree of selection effects by non-insurance of chronically affected US Americans cannot be estimated in a reliable way.

Due to the “Patient Protection and Affordable Care Act” passed by President Obama, there will be gradual changes in 2014, 2016, and 2018. So interested readers can expect future changes of the frequency of the diagnosis of “sinusitis” reported by the CDC/NHIS.

### 2.2 Prevalence of chronic rhinosinusitis (CRS)

Table 4 (Tab. 4) summarizes the most important publications of epidemiologic data of CRS, sorted by geographic regions. The cohort and the methods of data collection are also mentioned.

The only use of questionnaires is submitted to a standardized evaluation, especially regarding the use of nasal endoscopy [5] and/or imaging [6], and leads to relatively high frequencies of CRS. Under epidemiologic aspects, the data collection in the representative population (see Table 4 (Tab. 4): “population” as indication of the method) is generally considered as gold standard.

An increasing prevalence during the last years and significant regional differences of the incidence make it difficult to assess the data from the German perspective [7]. Even in the USA a geographic variation was reported: in the south of the USA, CRS seems to occur more often than in the north [8]. Investigations from Korea are important in this context: they show a clear increase in 2008 (7.12%) [9] compared to 1991 (1.01%) [10]. However, this can also the explained by the method applied: in the more recent investigation, residents and improved video endoscopes were used enabling nasal endoscopy to identify before and after decongestion smaller quantities of secretion or polyps. Further the clinical definition of CRS was changed from originally 3 necessary symptoms (nasal obstruction, smelling disorder, headaches or pressure, or rhinorrhea) to 2 symptoms (nasal obstruction and nasal secretion).

For Europe, a prevalence of CRS of 10.9% (95% CI: 6.9–27.1) is confirmed [11]. The evaluation was based on the definition of the European Rhinologic Society (ERS; http://www.europeanrhinologicsociety.org) [12] for the age group of 15–75 years.

Methodically, this investigation showed a high reliability of patient-related data for identification of CRS. This fact emphasizes the value of other, earlier evaluations from all mentioned tables of this review article that are only based on questionnaires and thus indicates an increased prevalence of CRS [11]. The correspondence of questionnaire-based and examination-based data of the prevalence was meanwhile described as sufficient (kappa 0.63) according to a pilot study from Sao Paolo [3]. 

For Germany, the guideline entitled “Rhinosinusitis” reports with reference to data collected by the Institute of Medical Statistics that in 2002 the diagnosis of chronic sinusitis was made 2.6 million times and the about 2.2 million patients per year seek medical assistance for chronic rhinosinusitis which leads to 3.4 million prescriptions [13].

European investigations show incidences for the regions of Duisburg (14.1; 95% CI: 2.0–16.6) and Brandenburg (6.9; 95% CI: 5.8–8.2) with low prevalence in non-smokers (11.9 and 5.3% respectively). It must be criticised that the return rate in Duisburg was low with 23.2% and that a significantly higher CRS prevalence was found compared to the average in Europe (10%). In Duisburg as well as in Brandenburg, the physician-based prevalence was significantly lower with 8.4% and 4.6% respectively, compared to a symptom-based diagnosis based on EPOS3 criteria (14.1% and 6.9%, respectively). Because of the fact that Brandenburg had a significantly lower CRS incidence than the European average, there seems to be a clear regional influence within Germany. Beside environmental influences (a higher CRS prevalence was observed in urban regions all over Europe) there may also be regional aspects as in rural regions such as Brandenburg ENT specialists are scarcely available. The differences in the industrial development, the nutritional status, and the living conditions during the time of the division of German must also be mentioned as possible factors.

### 2.3 Special subtypes of CRS

#### 2.3.1 CRS with and without nasal polyps

CRSwNP is defined as CRS with identification of nasal polyps. This identification can be reliably performed by means of nasal endoscopy and/or imaging techniques. Unfortunately, nasal endoscopy which is associated with low stress for the patient is used only in few population-based studies [14].

Already in 1977, the prevalence of CRSwNP was calculated with 4.2% in a hospital cohort, in asthma patients even with 6.7% [15]. In this investigation, the prevalence increased in higher ages [15]. The average age of the patients was 42 years [16], [17], [18]. First serial evaluations in cadavers showed nasal polyps in 2% [19] and 5 of 19 [20] and 13 or 31 cadavers [21].

Based on these data, the conclusion was drawn for England that in the course of a life 0.2–1% of the population develop nasal polyposis [22]. Symptomatic CRSwNP was given with an incidence of 0.86 for males and 0.36 for females per thousand in Scandinavia [23]. In this context, a increased rate was reported for higher ages up to 1.68 (♂) and 0.82 (♀) patients, respectively, per thousand and year in the group of 50–59 year-old people.

With reference to the symptoms, a differentiation between CRSwNP and CRSsNP is only inadequately possible. The most frequent subjective complaints in cases of CRSwNP (collected with the SNOT-22) are nasal obstruction [24] in 96.5%, disturbed smelling/tasting in 90.3%, and the necessity of blowing the nose in 79.8%. Furthermore, a runny nose (69.6%), viscous nasal secretion (66.6%), and otalgia (17.1%) were major complaints [24]. In cases of CRSsNP nasal obstruction is also the key symptom. More rarely, smelling/tasting disorders (75.5%), fatigue after waking up (69.7%), facial pains (69.7%), and posterior rhinorrhea (67.8%) were observed. Otalgia (35.3%) was reported more frequently than in CRSwNP [24].

The methodically best European investigation reports identification of CRSwNP by means of nasal endoscopy in 2.7% of the Swedish population [14] (Table 5 (Tab. 5)). Males (2.2; OR: 2.7 [95% CI: 1.33–5.5]), elder people (>60 years; 5%), and asthmatics (OR: 5.2 95% CI: 2.48–10.89) were more frequently affected [14]. Because of the fact that the examination was performed without endonasal decongestion (e.g. in contrast to the Korean investigation [9]) the prevalence of CRSwNP might even be higher in Europe than in Asia. The increase of CRSwNP in higher ages could be confirmed for Europe [14], the USA [15], and Asia [9]. Those investigations allow an interesting insight into the health care situation of European patients suffering from CRSwNP.

In Sweden, an advanced nasal obstruction and smelling disorder lead to the decision to seek medical assistance in cases of CRSwNP [25]. Among the Swedish patients with CRSwNP, 29% of the study population (corresponding to 0.8% of the population) knew about the diagnosis. So it seems that in Sweden only a clear minority of the population calls for medical aid for CRSwNP. In comparison to those data, the French population seems to be better cared for: 145/212 of the CRSwNP patients had been examined by a physician which led to a transferal to a specialist in 77.2% of the cases [26]. Medication was applied in two third of the patients and was still continued at the time of investigation. Surgery had already been performed in 13.4% of the French patients with CRSwNP which was completed by cortisone-based therapy in 27.7% of the cases. 92% of the French people who had undergone therapy for CRSwNP were satisfied with the results.

Further 1.4% of the Swedish population stated that they had suffered from CRSwNP earlier and that currently no endonasal polyps were detected [25]. The conclusion may be drawn that in about two third of the CRSwNP patients the treatment is successful in a singular sample.

Comparing the applied methods it can be said that the increase of CRSwNP observed in Korea from 0.5% ([10]; 1996) to 1.53% [9] is due to technical reasons as already described for the data of CRS.

In comparison to the evaluations using nasal endoscopy, questionnaire-based evaluations tend to show higher prevalence rates (Finland: 4.3% [27]; France: 2.1% [26]). Hence it seems to be appropriate to perform future questionnaire-based investigations, with applying nasal endoscopy whenever possible.

#### 2.3.2 Fungal sinusitis

Fungal sinusitis is classified into invasive and non-invasive types (based on the histology) as well as clinically acute and chronic courses of the disease. The chronic course requires symptoms persisting for more than 4 weeks [28].

A non-invasive, packed aggregation of fungal hyphae with inflammatory signs is called mycetoma. Often it is accompanied with mucous purulent secretion, without eosinophil mucin [29]. Typically it occurs as unilateral affection of the paranasal sinuses with calcification within the sinus in the CT scan of the paranasal sinuses [30].

A special importance is attributed to allergic fungal sinusitis (AFS). According to Bent and Kuhn [31] it is defined by the following symptoms:

- Radiological signs of sinusitis
- Detection of allergic mucin in the nose or paranasal sinuses with degenerated eosinophils with Charcot-Leyden crystals and branched fungal hyphae
- Detection of fungal hyphae in the allergic mucin
- Exclusion of invasive growth of the fungi by histology
- Exclusion of an immune deficit as for example in diabetes mellitus or therapy with immunosuppressants

In the USA, the vast majority of the fungal sinusitis is non-invasive (87.5% [32]. Significant regional differences are observed [32]: in southern states of the USA AFS is the reason for 10% of all paranasal sinus surgeries [33]. This percentage varies from 0–4% in the northern states to 23% in Memphis, Tennessee [33].

The subtype of AFS affects Afro-Americans in the USA more often (58%) than Caucasians (39%) [34]. A clear preference of the gender could not be observed [34], [35]. A lower income is associated with more severe disease. In 91% nasal endoscopy revealed an associated nasal polyposis [35].

Fungal parts can frequently be found in the paranasal sinuses in cases of CRS (Egypt: 92% [36]). AFS and the role of fungi is particularly important in scientific discussions. *Staph. aureus* could be detected more frequently in AFS patients than in other CRS subtypes (63.2 vs. 24.1%) [37].

Beside mucosal swellings with secretion the invasive fungal sinusitis is also characterized by hyphae in the mucosa/submucosa, bones, or vessels [28]. If granulomatous inflammation with giant cells is confirmed, the disease is called granulomatous invasive fungal sinusitis.

The invasive fungal sinusitis in its acute course of the disease has a mortality of 7.1% [38]. This severe type of sinusitis occurs in Africa, Asia, and the Indian subcontinent also in immunocompetent patients. It is caused by numerous pathogens, among others zygomycetes (rhizopus, mucor, rhizomucor) and aspergillus that are the most frequently detected species [38]. The delineation of the chronic course can be difficult in the individual case [38].

A clear majority of the literature in this context originates from Asia: fungal sinusitis in China [30] and India [39] is chronically invasive in about one third of the cases and non-invasive in about two third. In general a significant increase of the articles on this subtype is observed in the literature [39].

Retrospective investigations in India revealed fungal sinusitis in 42.7% of all RS patients having undergone surgery [40], [41] (Table 6 (Tab. 6)). Allergic fungal sinusitis is the main representative of the non-invasive type (Table 7 (Tab. 7)). However, the classification of fungal sinusitis used in Table 7 (Tab. 7) is still controversially discussed in scientific papers [32], [42], [43].

In comparison to CRS, fungal sinusitis in China affects more often female patients who are more than 40 years old. They report about suffering from the symptoms for less than three years that often occur as headaches and bloody nasal secretion [30]. In contrast, males seem to be affected more often in India according to the majority of the published studies (♂:♀=1.8:1 [40]; 1.33:1 [39]; 0.8:1 [44]).

#### 2.3.3 Dental sinusitis

Statements on the incidence of dental genesis of CRS are not possible because of missing data on the clinically rare diagnosis [45]. For the USA, 2.9 patients per year were estimated per ENT specialist or rhinologist [46]. Because of a hospital cohort, an increasing incidence is expected [47].

#### 2.3.4 Biofilms and MRSA

During the last years, the organization of different pathogenic organisms in biofilms was discussed as possible origin of CRS [48], [49]. The identification of biofilms in patients with CRSwNP is associated with a poorer prognosis after surgery [50], [51] and lower quality of life [52].

In revision surgeries, the detection rate is also increased (1.93; 95% CI: 1.01–3.69 [48], [53], [54]) as well as after previous treatments with topical steroids (2.09; 95% CI: 1.07–4.08 [53]). *Staph. aureus* seems to contribute significantly to the development of biofilms [55]. A colonization with *Staph. aureus* occurs more often in patients with CRSwNP than in healthy control groups [56]. Additionally, a reduced expression of lactoferrin is detected in cases of identification of biofilms [57].

Biofilms support the occurrence of resistances whereas especially methicillin resistant *Staph. aureus* (MRSA) has a high relevance in the context of health economics. Currently, the incidence of *Staph. aureus* positive cultures amounts to 7.7% and the one of MRSA positive cultures to 1.06% [58].

The incidence of nasal colonization with MRSA in the population is increasing: in a national survey in the USA the colonization increased from 0.8% (2001) to 1.5% (2004) [59]. Only 20% of the patients in whom MRSA was identified are permanent carriers. Further 20% are only rarely colonized (non-carriers), and 60% have a moderately frequent colonization with different strains [59]. The affection with (methicillin sensitive) *Staph. aureus* is currently decreasing according to further investigations: the detection in the USA of 32.4% in 2001–2002 decreased to 28.6% in 2003–2004 [59].

In this context, CRS with MRSA must be mentioned. Because of the clearly difficult MRSA eradication it is a particular challenge even if specific data is rarely found. Retrospectively, MRSA could be revealed in 9.22% of the patients with muco-purulent CRS [60]. Neither the number of earlier antibiotic therapies nor previously performed sinus surgeries have been described as risk factors. For Korea, MRSA identification was described in 4.75% after sinus surgery [61].

More recent evaluations revealed *Staph. aureus* in 19% of the patients with CRS [62]. Among those, 19% were MRSA so that in this context the incidence of MRSA in CRS is relatively stable with 3.8% of the CRS patients in the last years [58]. However, those data are not confirmed. Other investigations in hospitals report about MRSA rates of 1.8–20.7% [63].

With this background of only few data the problem of CRS with MRSA colonization should be dealt with more intensively in the future. This subtype is of special, also personal, importance for the treating physicians, the nursing staff, and the institutions because in single cases it may lead to inability to work of the people. So there is an urgent need for research in this field [64].

#### 2.3.5 Pediatric CRS

Because of the inclusion of the symptom of “cough”, pediatric CRS is defined in a different way than CRS in adult patients. Due to the low quality of the data and the reduced number of evaluations it is not possible to quantify the impact of this changed definition on the prevalence. So there are no representative investigations that include children. According to Settipane, 0.1% of the children in general and 20% of the patients suffering from mucoviscidosis are diseased with CRS [65].

Long-term evaluations of children are available, sometimes even with repeatedly performed X-ray imaging. Long-term changes of the paranasal sinuses occur in 30% of the children. With adolescence, this number decreases to 15% [66]. The problem in this context is the missing delineation of recurrent acute sinusitis and CRS, the high age of the study (75 years), and the poor sensitivity and specificity of the X-ray overviews of the paranasal sinuses. On the other hand, procedures with low radiation exposure alone are not helpful. Opacities in the MRI are observed in nearly 100% of all examined children [67], [68], [69], [70], while the majority of the children has not symptoms at all [71].

Because of the early age of manifestation of CRS, a genetic component was discussed repeatedly, especially in cases of missing other risk factors [72]. As genetic factor for CRS in children, single nucleotid polymorphisms (SNPs) for KCNMA1, a gene for potassium ion channels, were reported [73]. Also, a decreased vitamine D3-level has been discussed as risk factor in children [74]. However, no data are available on the prevalence in the population.

In a cohort of atopic children, the exposure to tobacco smoke turned out to be a risk factor for the development of CRS (OR: 3.96 95% CI: 1.50–10.48) [75]. Further, the presence of food allergies could be confirmed as risk factor (HR: 0.26 95% CI: 0.10–0.66, P=0.004) [75]. Finally, children with CRS seem to have more often a private health insurance and thus benefit from a higher economic standard [76]. It might also be possible that a better access to the health care institutions for those children is responsible for those data in contrast to children with a lower social status.

In summary, future representative investigations of children with CRS seem to be urgent. Up to the time when current rates are published, CRS in children must be considered as rare disease with a prevalence of 0.1% for CRSwNP [65].

#### 2.3.6 Disorders of mucociliary clearance

**CRS because of genetic predisposition. **CRS as consequence of a ciliary defect such as a primary ciliary dyskinesia or cystic fibrosis (mucoviscidosis) plays a particular role in children. The primary ciliary dyskinesia can also be observed in 5.6% of the children with recurrent infections [77]. In the population of south England, it occurs in 1:2,265 cases [78], the prevalence of patients with a Kartagener syndrome only in 1:32,000. Apart from the genetic origin of the disorder, CRS is developed more often in CRSwNP than in CRSsNP.

Cystic fibrosis has an incidence of about 1:2,500 with increasing tendency [79], [80]. In these cases, nasal polyposis was reported in 4–44% of the patients [81], [82], [83], [84], [85]. In the subgroup of those patients who had undergone lung transplantation CRSwNP was observed in 10% [86]. Inversely, patients with CRSwNP had a genetic mutation associated with cystic fibrosis in 6% [87].

Another genetic factor for the genesis of CRSwNP is mentioned in a Polish study that found an increased rate of a –765 G/C polymorphism of cyclooxygenase-2 (OF: 4.04; 95% CI: 2.32–7.03) and a C allel (OR: 3.68; 95% CI: 2.38–5.68) in comparison to the control group of the same age and gender [88]. Further the study reports about families that allow the conclusion of a still not clarified hereditary background of CRSwNP [89]. Possibly HLA-A74 plays a role in this context [90].

Two different polymorphisms in the field of tumor necrosis factor (TNF)-alpha protein 3 gene that are associated with severe CRS (rs3757173 OR: 1.67; rs5029938 (intron 1) OR: 1.95) [91]. Additionally, 3 single nucleotid polymorphisms of the IL22RA1 area showed significant differences regarding their occurrence in patients with severe CRSwNP in comparison to healthy individuals of the same region (rs4292900 P(nom)=0.0006, OR: 1.757; rs4648936 P(nom)=0.0011, OR: 1.716; rs16829225 P(nom)=0.0014, OR: 1.977) [92].

In China, another pathophysiology seems to be the origin of CRS according to extensive investigations performed by Bachert et al. (compare paper on the pathophysiology published in the same issue). An investigation of genetic influences for the development of CRS with RYBP (rs4532099, p=2.15E-06, OR: 2.59) and AOAH (rs4504543, p=0.0001152, OR: 0.58) could reveal factors that had not been reported for Europe up to now. In contrast, CRSsNP was associated with RYBP (*P*=3.24^E^-006, OR: 2.76) [93]. CRSsNP would then be (as already the general CRS) associated with a type of RYBP (OR: 2.45) and AOAH (OR: 0.3).

### 2.4 Prevalence of CRS in different human races

In the USA, there was no difference found in the prevalence of Caucasians and Afro-Americans from 1990–1992 (2% each) [2]. However, the postoperative result in Afro-Americans seemed to be poorer [94] and (as already mentioned) AFS occurs more often in Afro-Americans in the USA than in Caucasians [34]. Asians and the Hispanic part of the population are more rarely diagnosed with CRS [95].

In the Chinese population the prevalence of CRSwNP based on aspirin exacerbated respiratory disease (AERD) seems to occur more rarely than in Europe [96]. This percentage was estimated to amount to 0.57% of the patients with CRS while AERD was associated in 5.7% (95% CI: 4.4–7.1%) with the development of CRSwNP in 36 [65] to 96% [1] of the Finnish population [27]. As the Polish evaluation stated the prevalence of the population with 0.6% [97], the relatively low incidence of AERD may also be due to geographical variations of this subtype of CRS.

### 2.5 Differences of the gender in the prevalence of CRS

The majority of the studies reports about a slightly higher prevalence in the female gender (Table 3 (Tab. 3) and Table 8 (Tab. 8)). In contrast to that, an investigation performed in Korea identified the male gender as risk factor for CRS [98]. Possibly the observed differences are also due to longer duration of the disease in men. Boys had a poorer prognosis regarding the surgical therapy of pediatric CRS [99].

Unfortunately the duration of the CRSwNP of 22.4±15.7 years as reported by Klossek for France was not classified according to gender which could have been a hint to a missing significant difference [26]. On the other hand, the higher health awareness of women associated with a more frequent seeking for medical assistance could compensate a higher disease rate of men caused by a widespread smoking habit [11]. A detailed investigation from Germany revealed a comparable rate of medical consultation for both genders (4% of all medical contacts) regarding ENT specialists in an investigation that was not specific for CRS [100]. This is why the question of a significant gender difference must be answered negatively.

### 2.6 Influence of the age on the prevalence of CRS

In Europe, the prevalence of CRS decreases in the age group of >55 year-old patients (OR: 0.89; 95% CI: 0.81–0.98) [11]. There were no differences found in the age groups of the less than 35 year-old and 35–54 year-old patients. These data contrast to data reported from North America where the rate increases from 2% to 7% between the 18^th^ and 70^th^ year of life based on subjective complaints [2]. Retrospectively, an increase of CRSwNP observed in a US hospital population of 39.6% in the 16–59 year-old patients to 68.2% in the 60–77 year-old patients [101]. This deterioration could be objectivized by an increased Lund-MacKay CT score. The decrease of eosinophil cationic protein was explained as a hint to a changed pathophysiology of CRSwNP in higher ages. For Asia, an increased risk to acquire CRS in higher ages could be revealed for Korea [98]. 

Another US-American investigation showed a lower incidence of CRS beyond the age of 65 [8]. In Canada, an increase up to the age of 60 was observed with subsequent decrease of the incidence of CRS [102].

While the differences described in this paragraph may be due to regional effects, the higher prevalence of younger patients may be a hint to an increasing total incidence of CRS in Europe in the future.

## 3 Comorbidities associated with CRS

### 3.1 Bronchial asthma and COPD

Bronchial asthma is a chronic inflammatory disease of the airways that leads to a reversible bronchoconstriction with increased production of mucous secretion. The key symptom is a sudden attack of dyspnea with wheezing expiration sounds. In the literature, the types of asthma are classified as allergic (extrinsic) and non-allergic (intrinsic) asthma. Similar to CRS, the different groups are generally not considered in epidemiologic observations [103]. Chronic obstructive pulmonary disease (COPD) is also a collective term for diseases of the lung that are characterized by cough, sputum, and dyspnea under stress.

According to a large European multi-center study, CRS is not automatically associated with a deteriorated lung function [104]. Because of a less important decrease of the pulmonary function in higher ages of patients with asthma and CRS, the authors even discuss a possibly protective effect [104]. Unfortunately, CRS was not differentiated into the subtypes of CRSwNP and CRSsNP.

For the German population, a survey revealed a prevalence of asthma of about 5.4% [103] which is higher for females (5.9%) than for males (4.8%). In this context, the age structure is different in the genders. In females the prevalence increases consistently with the age whereas males acquire the disease up to the 64^th^ year of life in only 4%, the prevalence at higher ages increases to 6.8% [103].

In Finland, about 4.4% of the population suffer from asthma (95% CI: 3.3–5.5%) and 3.7% from COPD (95% CI: 2.7–4.8%) [27]. In the same cohort, the prevalence of CRSwNP amounted to 4.3% (95% CI: 2.8–5.8%). According to the questionnaire-based analysis, nasal polyposis occurs nearly as often as bronchial asthma and more often than COPD.

A strong relationship between CRS and bronchial asthma could be confirmed for all age groups in the GA2LEN study (adjusted OR: 3.47; 95% CI: 3.20–3.76) [105]. With simultaneous bronchial asthma, the risk of acquiring CRS amounted to 1.94 in men (95% CI: 1.24–3.03) and to 1.73 in women (95% CI: 1.36–2.20) in Canada [102].

In a Swedish subgroup of the GA2LEN study, a clear reduction of the quality of life as well as a poorer pulmonary function could be revealed with simultaneous presence of CRS and bronchial asthma [106]. An additional reduction of the quality of life of asthmatics was also reported for CRSwNP [107] and RS [108]. Especially smell in this combination is impaired and can be considered as indicator for the severity of the disease [109]. In contrast to asthmatic children [110], adult asthmatics with CRS suffer more frequently from an acute deterioration of asthma [111], [112], [113].

45% of CRS patients in France [114], [115] and 71% [15] of the CRS patients of an American hospital population suffer from bronchial asthma. Also the Brazilian investigation [3] showed a higher prevalence of asthma in the group of the CRS patients (OR: 3.88; 95% CI: 1.94–7.77%). Even in patients with recurrent CRS, asthma is found more frequently [116]. Hence an association between CRS and bronchial asthma is confirmed on a worldwide scale.

Data on the association with subtypes of CRS are very rare and primarily concern CRSwNP: 7% [65] –15% [117] of all asthmatics also suffer from CRSwNP. Prospectively, bronchial asthma could be revealed in CRSwNP patients more often (23% [26]; OR: 5.9; 95% CI: 1.79–19.65 [118]) than in the control group (6% [26]). An influence of atopy could not be confirmed neither on CRSwNP nor on asthma [118]. Women with CRSwNP develop 1.6 times more often bronchial asthma [119].

Based on the clinical symptoms, 53% of the COPD patients suffered from CRS [120]. Computed tomography could even reveal CRS in 64% of this population. The majority of the COPD patients has suspicious findings in nasal endoscopy, among others also nasal polyposis [121].

Beside CRS, bronchial asthma and COPD are associated with other diseases that are also discussed as risk factors of RS. Those are for example aspirin exacerbated respiratory disease, gastro-esophageal reflux [112], [122], [123], [124], obesity [125], [126], and depression [125], [127], [128]. Hence, the characteristic association or the common pathophysiology of CRS and bronchial asthma may also be responsible for these and further, newly described associations.

### 3.2 CRS and atopy/allergic rhinitis

Because of the enormous incidence of both diseases, the location in the airways, and the combined inflammatory pathophysiology, also a synergistic effect of CRS and allergy was repeatedly discussed.

The relatively high prevalence and incidence of allergic rhinosinusitis and CRS make it difficult to analyze the interaction: the prevalence of allergic rhino-conjunctivitis was reported with 10–68% [129], [130] (Canada: 17% [131], Finland: 37.3% [95% CI: 33.3–41.2%] [27]) while an increased prevalence in CRS patients is still controversially discussed [1].

Considering population-based investigations, positive prick tests are reported in 50–84% of the patients [132], [133], [134], [135]. A positive prick test, however, does not increase the probability to suffer from CRSwNP [15], [136]. According to that, the European position paper classified the prevalence of allergic rhinitis in CRS patients as increased [1]. Studies performed in Sao Paulo and Belgium could not reveal a relationship between sinusitis and chronic rhinitis (without the differentiation if allergic genesis was present) [3], [137]. In Korea, the allergic rhinitis is described as most important risk factor of CRS (OR: 8.23; 95% CI: 4.70–14.43) [98].

Patients describe a positive effect of desensitization regarding acute exacerbations [138] and confirm improved symptoms after immunotherapy [139]. The improvement in this context rather concern recurrent acute courses of the disease than CRS [138].

As the incidence of CRS during the pollen season neither increases or decreases, a causal association is still questionable despite possibly higher prevalence [1], [140]. Alternatively, a higher prevalence may be due to improved examination rates of allergy sufferers [141], [142], [143].

The prevalence of CRSwNP in allergy sufferers amounts to 0.5–4.5% and is comparable to the general population [15], [144], [145]. With 2.2% it was even observed more rarely in patients with allergic rhinitis than in asthmatics (6.7%) [15]. Asthmatics without atopy had more rarely CRSwNP than asthmatics with atopy (12.5 vs. 5.0%) [15]. In contrast, allergic rhinitis could be increasingly detected in recurrent CRS [116].

Food allergies seem to play a special role in this context. Positive prick tests for food allergens were found significantly more frequently in patients with CRSwNP (70%; 81%) compared to control groups (34%; 11%). Although prick tests are considered as technically not reliable for food allergies, questionnaire-based investigations (22% [26]; 31% [114]) and intradermal tests (81% vs. 11%) support a more often occurring food allergy of patients with CRSwNP [146].

Finally, the role of allergies as impact factor of CRS remains controversial [1], [147]. Possibly, the Asiatic pathophysiology of CRS is different from the European type because of a synergism with the allergic rhinitis. Allergy diagnosis and therapy if needed, however, are a further therapeutic option for CRS patients [1].

### 3.3 Aspirin exacerbated respiratory disease (AERD)

Specific side effects of aspirin were first described by Hirschberg in 1902 [148], the full scope of aspirin exacerbated respiratory disease (AERD) was analyzed by Widal in 1922 [149], and in the English speaking countries in 1968 by Samter and Beers [150]. Regarding the pathophysiology, the inhibition of the enzyme cyclooxygenase 1 leads to a shifting of the arachidonic acid metabolism with increased activity of 5-lipoxygenase by non-steroidal anti-inflammatory drugs (NSAID). This leads to an increased production of pro-inflammatory leukotriene that seem to be responsible for the consecutive effects with inflammatory diseases of the upper and lower airways.

After initial complaints of rhinitis, the usual course of AERD includes the occurrence of bronchial asthma after two years, followed by an intolerance of NSAID after further two years [151]. In comparison to other European countries, symptoms of rhinitis are more often found in Germany (90%) [151].

On the average, AERD patients are 44.7±14.25 years old, mostly female (57% [152]; 76.6% [151]) and in their third decade of life at the time of first diagnosis. The female gender is also associated with an earlier beginning and more severe course of the disease [151]. In 6% of the patients a positive family history is observed [151].

The incidence of AERD for the general population is estimated to 0.3±0.6% [27], [97], [153] (Table 9 (Tab. 9)). AERD manifestation of the lungs was observed in 1.2% of the Finnish population [27]. In Australia, those values amount to 2.5% [154] and in the general Finnish population even up to 5.7% (95% CI: 4.4–7.1%) [27]. In summary, up to 6% of the European population suffer from AERD.

Beside atopy (OR: 2.80; 95% CI: 1.38–5.70) and the number of asthma attacks during the preceding year (OR: 1.20; 95% CI: 1.02–1.42) also the proof of CRSwNP (OR: 3.39; 95% CI: 1.57–7.29) count among the independent predictors of AERD [154].

In about one third of the patients, atopy is present [151] and thus it occurs more often than in a control group of the same age [155]. Allergies are revealed even in 30–60% of AERD patients by means of prick or intradermal tests [151]. They are associated with an early manifestation of rhinitis and bronchial asthma [151].

Asthmatics in Poland suffer from AERD in 4.3% (95% CI: 2.8–5.8) which is more often than in the general population [97]. In Finland, AERD was found even in 8.8% of asthmatics (compared to 0.8% of non-asthmatics; relative risk 11.4) [27]. In Australia, AERD was found in asthmatics in a hospital in 10.7% of the cases (95% CI: 5.8–15.6) and in patients treated on an outpatient basis in 10.4% (95% CI: 7.3–13.5) [154]. A randomized study of the Australian population confirmed an AERD prevalence of 10.9% for asthmatics in general [154]. 

The variation of the prevalences can be explained by the different investigation protocols. When a provocation test together with spirometry was applied, AERD was observed in 8–20%. The questionnaire-based prevalence is lower with about 5% [27], [153], [156], [157]. Review articles report about an incidence of 21.1% (95% CI: 13.6–28.6) of AERD in asthma patients that even increases to 38.7% (95% CI: 33.2–44.2) when a provocation test is performed [158]. A positive history in an unselected patient population, however, is only associated with the proof of AERD in asthmatics in 2.7% (95% CI: 1.6–3.8) [158]. According to the AIANE study up to 80% of AERD patients need high doses of inhalation steroid [151]. Additionally, 51% of the patients systemically apply cortisone [151] in order to control the bronchial asthma.

In summary, adult asthmatics suffer from AERD in 3–38.7%. The association of AERD increases to 24% of the patients in cases of severe asthma and in cases of asthma with CRSwNP to 40% of the patients [27], [97], [158].

In Europe, 66.7% of AERD patients stated to suffer also from CRS [97] and in 60% CRSwNP was confirmed by nasal endoscopy [151]. The prevalence of CRS even increased to 90% in AERD patients after CT scan of the paranasal sinuses [151]. In Australia, CRSwNP could be found more rarely, i.e. in 31–34% of AERD patients, according to an interview [154].

Within the group of CRS patients, AERD is diagnosed too rarely [97], [159]. AERD prevalence in CRS patients amounted to 4.8% in the USA [160] and 9.4–15% in CRSwNP [15], [160], [161] and was thus superior to the prevalence in asthmatics (3.8%) or in rhinitis (1.4%) [153].

The incidence and associations of AERD, bronchial asthma, and CRS/CRSwNP have important regional differences.

#### 3.3.1 Hints on different phenotypes within AERD

Regarding the genesis of AERD, there are new hints for genetic as well as environmental influences:

With reference to the pathogenesis of AERD, data from Korea reveal a genetic component in the field of FSIP1 (fibrous sheath interacting protein 1 gene) polymorphism on chromosome 15q14 in the comparison of AERS with aspirin tolerant asthmatics (R7179742; OR: 1.63; 95% CI: 1.23–2.16) [162]. Further genetic polymorphisms with association of AERD concern the adenosine A1 receptor [163], the prostaglandin and thromboxane receptors [164], the promoter of cysteinyl leukotriene receptor 1 [165], interleukin 10 [166], angiotensin I converting enzyme [167], and the Fc fragment of immunoglobulin E [168].

For the occurrence of AERD (OR: 3.46; 95% CI: 2.22–5.39) tobacco smoke exposure in childhood was identified as risk factor [169]. AERD patients in this evaluation were also active smokers (OR: 1.54; 95% CI: 1.04–2.28), however, the combination of tobacco smoke exposure as child and adult further increased the risk of AERD (OR: 5.09; 95% CI: 2.75–9.43) [169]. If this parameter is also confirmed for Germany, a significant increase of AERD could be expected for every second child because of the tobacco smoke exposure revealed by Robert Koch Institute [170].

### 3.4 Eosinophil granulomatosis with polyangiitis (EGPA, formerly: Churg Strauss Syndrome)

Another important subgroup of patient with CRS are those who suffer from sinusitis as part of an eosinophil granulomatosis with polyangiitis (EGPA). This disease was formerly known as Churg Strauss Syndrome and according to the American College of Rheumatology [171] it can be confirmed with high specificity and sensitivity if 4 of the following 6 manifestations are present:

- Bronchial asthma
- Eosinophilia >10%
- Sinusitis (anamnestically acute or chronic; alternatively also opacity in imaging)
- (if applicable transient) pulmonary infiltrates
- Histologically confirmed vasculitis with detection of extravascular eosinophils
- Mononeuritis multiplex or polyneuropathy.

For northern Germany a prevalence of EGPA was reported with 9.0 per million person years (MPY) [172]. This corresponds approximately to data from France (10.7; (95% CI: 5–17)/MPY [172]), England (6.8 (95% CI: 1.8–17.3)/MPY [173]), and Sweden (14 (95% CI: 0.3–27)/MPY [174]). Data from American studies on the incidence of EGPA in asthmatics vary between 0 (90% CI: 0.0–23.0) and 67 (90% CI: 22.5–160.6) patients per MPY [175]. The range of variation can be explained by the application of different definitions of EGPA [175].

Men are less frequently affected than women (1.12:1) [176]. The age of the patient at the time of first diagnosis amounts to about 50 years [177].

In more than 80% the patients complain about nasal symptoms [178]. Sinusitis can be confirmed in the majority of the patients (77.4% [179]) with EGPA without the aspect that pathological antinuclear cytoplasmatic antibodies (ANCA) change the incidence of sinusitis (ANCA+: 77.1; ANCA-: 77.6%) [179]. CRSwNP is present in about 60% [180]. About 20% of the patients undergo surgery of the paranasal sinuses before diagnosis of EGPA and 40% undergo other rhinosurgical interventions [178] without an association of the sinusitis with a severe course of EGPA [181].

Currently sinonasal symptoms lead to the diagnosis of EGPA in 28% [178]. This percentage is only exceeded by asthmatic deterioration (40%) which means a special responsibility for ENT specialists and rhinologists regarding the confirmation of the diagnosis of EGPA. The particular problem for the ENT specialist is the large subgroup of patients with CRS and bronchial asthma who also have an eosinophilia >10%. 

Regarding the pathogenesis, diagnostics, and therapy we refer to another paper in this issue entitled “Orphan diseases of the nose and paranasal sinuses: Pathogenesis – clinic – therapy” by PD Dr. Martin Laudien [182].

### 3.5 Helicobacter pylori and gastro-esophageal reflux (GERD)

DNA of *Helicobacter pylori* was found in 11% [183] to 33% of the patients with CRSsNP. According to an extensive review [184] the indications up to now are not sufficient to classify gastro-esophageal reflux as origin for the development of CRS or to recommend an appropriate therapy [1]. In the USA, however, because of the simultaneous occurrence of both diseases in 45% of the examined patients, this enormous incidence was characterized as more than a coincidence [185].

In children with CRS, this aspect seems to be clearer: American children with RS have a high prevalence of GERD (63%) [186] that exceeds the one of a control group (4.19 vs. 1.35%) [187]. According to an investigation from Turkey, children with CRS had also a reflux in 38% of the cases [188]. Drug therapy for reflux (among others by means of proton pump inhibitor) can improve the symptoms of CRS in 79–89% of the cases [186], [189], [190]. In single cases, also a surgical reflux therapy was successful with regard to CRS [191].

Because of the limited data, the European position paper does not consider reflux therapy as standard option [1].

### 3.6 Chronic headaches and CRS

Based on representative surveys of the population, the prevalence of chronic headaches (>15 days per year) is estimated to 2.14% in Norway. The complaints of 0.33% of the total sample or 15.3% of the headache patients could be confirmed as originating from CRS [39]. Thus, CRS is the second most common genesis of chronic headaches after drug-induced headaches [39].

## 4 Selection of possible risk factors for CRS

### 4.1 Sinonasal anatomic variations

Traditionally, anatomic variations are expected to be risk factors for CRS and surgical correction is recommended. Regarding the definition we refer to the European position paper on the terminology [192].

After critical analysis of the literature, it can be stated that a slight septal deviation [10], [193] has no influence on the incidence of CRS while deviations of >3 mm are associated with a higher prevalence in some investigations [194], [104], in others, however, they are not [193], [195], [196]. Accordingly it can be explained why a Korean study did not classify septal deviation as risk factor [10] whereas a more recent study in the same country came to a contradictory conclusion [98].

Nearly the same distribution of CRS patients and healthy people was described for a paradox bending of the uncinate process [197], agger nasi cells [197], [198], spheno-ethmoidal or Onodi cells [195], the configuration of the rhinobase [195], [197], or the size and form of the ethmoidal bulla [198]. Accordingly, anatomic variations were often not valuated as causal factor for CRS [195], [198], [199], [200], [201].

Contradictory data are given for concha bullosa (33 vs. 11% [202]; 73 vs. 78% [197], [203]). The same is observed for infraorbital cells (Haller cells) (HR: 7.39 [197], [204], [205]). Also for frontal inter-sinus septal cells (HR: 68.03) there are hints for an increased risk of CRS in patients with allergic rhinitis [204].

Because of investigations performed in our department [206] it must be stated that up to now only the proof but not the extent or volume of the mentioned variations has been analyzed. Representative comparative values collected in the population are still missing. Finally, methodically only a slight correlation in the assessment of the singular anatomic variations was found among the different examiners of the CT scans [207]. Hence, this risk factor cannot be finally evaluated.

### 4.2 Smoking and exposure to tobacco smoke

In 2009, 21% of the American population stated that they were active smokers, other 21% had given up smoking [95]. Germany exceeds those values: in Germany men and women smoke much more. The percentage of men who smoke is between 29% (upper class) and 47.4% (lower class) and the percentage of women who smoke is between 25% (upper class) and 30.1% (lower class) [170].

In Europe, an association between smoking habits and the occurrence of sinusitis was confirmed (OR: 1.91; 95% CI: 1.77–1.05) [11]. For females this association could be confirmed in Canada (OR: 1.57; 95% CI: 1.24–1.99) [102]. Hereby the risk is increased for active smokers (OR: 1.67; 95% CI: 1.18–2.37) and for former smokers (OR: 1.20; 95% CI: 0.90–1.60) [102]. This risk even increases if an allergy is found additionally, for active smokers to an OR of 1.41 (95% CI: 1.02–1.96) and for former smokers to an OR of 1.24 (95% CI: 0.91–1.69%) [102]. Although this negative effect could not be proven in Brazil [3] and men in Canada (adjusted OR: 1.24; 95% CI: 0.9–1.7%) [102], the majority of the investigations indicates exposure to tobacco smoke as risk factor for CRS. In this context also passive smoking is relevant [208], especially for children [209].

Air stewards as occupational group are often exposed to passive smoking. Interviews of more than 2,000 air stewards revealed that 43.4% suffered from RS. This prevalence increased with the exposure to passive smoking [210]. An intensive scientific discussion was caused by a retrospective evaluation in this context that identified smoking as risk factor for the development of nasal polyposis in the USA, at the same time it came to the conclusion in a regression analysis that heavy smoking could protect against nasal polyposis [211], [212], [213], [214]. The authors mention a reduced immunoreaction caused by smoking as explanation, however, they also admitted possible methodical influences such as the sample size.

Methodical problems, however, may also be caused by the fact that smokers without CRS achieve higher values in specifically rhinologic questionnaires (e.g. SNOT [215]) than non-smokers. So there is the possibility that merely questionnaire-based assessments in the group of smokers systematically overestimate the prevalence of CRS.

### 4.3 Occupational and environmental influences

There are hints indicating that special occupational groups more often develop CRS because of environmental influences.

A Danish subgroup of the GA^2^LEN study reports about a higher prevalence for female blue collar workers in comparison to white collar workers (employees and self-employed people) [216]. For men, this difference depended additionally from their smoking behavior. Especially exposure to dust, gas, smoke, and steam were classified as being responsible for disease.

Data from Lower Saxony confirm a higher percentage of diseases of the lower airways in close neighborhood to large fattening farms [217]. Despite nasal symptoms that have also been revealed increasingly, the data situation is too poor to be described as increased prevalence of CRS [218], [219], [220].

In the USA, 7–9 years after the service at the World Trade Center a higher prevalence of sinusitis (9.1%) was assessed in fire brigade forces [221] which developed parallel to a functional deterioration of the lower airways. In Canada, a clearly increased risk to develop CRSwNP (OR: 13.1) was found in patients heating with wood [222]. For employees in plant industries, engineering, and installation as well as crafts, Korea reported a higher prevalence of CRS [223]. The same was true for unemployed people. The interpretation of those data is problematic because even a lower socio-economic standard is associated with increased complaints of CRS [224] and this relationship could also alternatively explain for example the effects of the Canadian heating behavior [222].

## 5 CRS as comorbidity in special primary diseases

### 5.1 CRS and cardio-vascular diseases

Chronic inflammatory processes seem to be associated with a higher risk of cardio-vascular diseases. Today, conventional risk factors can only explain 50–75% of the cardio-vascular events [225].

#### 5.1.1 Acute myocardial infarction

The incidence of acute myocardial infarction is increased to 8.49 in patients with RS in comparison to the population of the same age with 5.09/1,000 person-years [226]. Further a higher incidence was revealed in RS patients (6.19/1,000 person-years; 95% CI: 5.01–7.65) in comparison to 3.51/1,000 person-years (95% CI: 3.06–4.02) in the control group [227]. Thus the hazard ratio during the 6 years of follow-up in RS patients was 1.70 (95% CI: 1.52–1.91). The higher risk was present despite the consideration of other cardio-vascular risk factors for patients with CRS ([226]: HR 1.70; 95% CI: 1.52–1.91; [227]: HR 1.78; 95% CI: 1.37–2.32). However, the CRS patients of the Taiwanese cohort mostly lived in urban regions and had a higher monthly income. Myocardial infarction occurred in particular in the first year after diagnosis of CRS (44.7%), accordingly, the risk decreased with every year after diagnosis of CRS (2^nd^ year: 40%; 3^rd^ year: 15.3%).

Differentiating those data on RS with regard to the acute or chronic course of the disease, there is a higher risk for acute rhinosinusitis patients to develop an acute myocardial infarction (HR 2.09; 95% CI: 1.03–4.14) [227].

#### 5.1.2 Apoplexy

In this context, investigations from Taiwan describe a higher risk for CRS patients to suffer a stroke. According to that, the hazard ratio amounts to 1.34 (95% CI: 1.04–1.74) [228]. This relatively low increase of the risk was confirmed in a second investigation for an ischemic cerebral event [229]. During the 5-year follow-up those patients had a stroke after diagnosis of CRS in 10.65 (95% CI: 9.93–11.41)/100 person-years in comparison to 7.53 (95% CI: 7.18–7.89)/100 person-years. No effect of intracerebral bleedings could be detected for CRS patients but only of ischemic events (HR 1.34; 95% CI: 1.18–1.53).

### 5.2 Obesity

In the USA an increased incidence of obesity was observed in CRS patients (OR: 1.31; 95% CI: 1.18–1.45). Applying the body mass index, the increase was significantly associated with the occurrence of CRS (OR: 1.022) [230]. In a Taiwanese population-based study, obesity (adjusted OR: 2.50; 95% CI: 1.90–3.30) but also loss of weight (adjusted OR: 2.58; 95% CI: 1.30–5.13) was described more often in CRS patients [231].

### 5.3 Depression

Patients with CRS tend to develop more easily psychological/psychiatric diseases. CRS patients possibly suffer more often from depression. This additional diagnosis was found in 26% of the CRS patients of a retrospective investigation [232]. In Canada, the additional diagnosis of depression was made in more than twice as much (8.4 vs. 4.1%) and also the antidepressive therapy with drugs was performed (9.1 vs. 4.6%). Further, psychological therapy is more often prescribed in patients with CRS (11.8 vs. 7%) [233]. On the other hand, anxious and depressive patients are more sensitive regarding CRS symptoms [234].

### 5.4 Immunosuppressed patients and CRS

#### 5.4.1 Deficient immune defense

In cases of therapy-refractory CRS an immunodeficiency was detected in a relevant part of the patients (22% [235]–55% [235]). This may also explain an increased number of bacterial microcolonies in the mucosa as possible pathogenetic co-factor of CRS [236]. The lack of immunoglobulin G (18% [235]; 9% [237]), A (17% [235]; 3% [237]), and M (5% [235]; 12% [237]) was reported [238]. A variable immunodeficiency syndrome was confirmed in up to 10% [235], [239] and in Europe and North America it is the most frequently occurring immunodeficiency with an incidence of 1:25,000 and 1:66,000. CRS can be identified in 36–78% of the patients with this immunodeficiency. In this subgroup, often anergy is found, too [235], or a sensitization in the allergy test [235], [236], [237], [238] and a reduced anti-pneumococci titer [237], [238]. Vice versa, patients with a reduced immune-response develop CRS in 77% after vaccination against pneumococci [240]. Further a reduced function of the natural killer cells was found in therapy refractory CRS [241].

#### 5.4.2 HIV/AIDS

34–54% of HIV positive patients show sinonasal symptoms in the sense of RS [242] which is significantly more often than in the control group. CRS was given with 34% in spain [243] and 12% in Brazil [244]. At the time of outbreak of AIDS, the CT-based Lund-MacKay score was significantly lower than in the single presence of HIV infection [244]. Accordingly, an African control study could reveal HIV/AIDS as independent factor for the occurrence of CRS (OR: 19.6; 95% CI: 4.3–88.9) [245]. In children diseased with HIV, CRS prevailed in 7.8%, however, in this study the symptoms had to persist only for 4 weeks in order to be classified as CRS [246].

So CRS in HIV/AIDS seems to be slightly more frequent than in immunocompetent patients. Data from Europe are missing.

#### 5.4.3 CRS and transplantation

There are data on patients before liver transplantation who suffer from CRS in 2.8%. Thus CRS does not seem to occur more often than in the general population. After transplantation, no complications of CRS occurred, however, the patients received antibiotic therapy for 4–6 months. CRS worsened in 4/22 patients. Hence, the authors did not give a general recommendation for sanitation surgery [247].

#### 5.4.4 Invasive fungal sinusitis in hematologic diseases

The incidence of invasive fungal sinusitis in Taiwan slightly reduced from 2.2% (1995–1999) via 1.63% (2000–2004) to 1.62% (2005–2009). For patients with invasive fungal sinusitis, AML (OR: 5.84; 95% CI: 1.02–30.56) and a therapy refractory leukemia (OR: 4.27; 95% CI: 1.003–18.15) could be identified as negative prognostic marker. In contrast, a surgical intervention was a positive prognostic factor for patients with invasive fungal sinusitis [248].

The prevalence of invasive fungal sinusitis during induction chemotherapy in cases of acute myeloid leukemia amounted to 8.9% in Singapore [249]. The acute lymphatic leukemia seemed to be less affected with a prevalence of only 1% [249]. In comparison to that, the prevalence of invasive fungal sinusitis in patients receiving allogenic bone marrow transplantation amounted to 16.1%.

The mortality in hospitals caused by invasive fungal sinusitis (and not by the basic disease) was 12.8% in this cohort of hematologically diseased and immunosuppressed patients [249]. The risk to develop invasive fungal sinusitis was significantly increased in cases of neutropenia of >10 days [248]. CRS was also found to be a risk factor for a fungus induced osteomyelitis of the skull base. In the present article, however, the subgroup of CRS was not exactly defined [250].

In summary, further immunological investigations of severe courses of CRS must be performed. The risk of CRS for patients that might be immunosuppressed in the future, however, is considered as being rather low.

### 5.5 Further associations

An investigation from Taiwan indicates a possible increase of the incidence of CRS in patients with cataract (5.16 vs. 3.45%) [251]. Current studies performed in Taiwan report about a 3.55 fold increased risk of CRS patients to develop nasopharyngeal carcinomas (HR: 3.55; 95% CI: 2.22–5.69) [252] and an OR of 3.83 (95% CI: 3.23–4.53) [253]. The population-based study published by Chung found a significantly increased adjusted OR for the group of CRS patients in 27/39 diseased people. This high frequency relativizes the clinical importance of those literature reports on the one hand, but on the other hand it indicates the scientific potential of such data. However, from a German perspective, the associations of CRS can only be understood as suggestion and must be critically discussed.

## 6 Economic significance of CRS

The following paragraphs will present cost analyses from the USA as well as current incidences of cases and procedures from Germany in order to give and overview of the economic dimension of the treatment of CRS.

### 6.1 Data on RS

In 1996, 5.8 billion US$ were already spent for the treatment of RS, of which 1.8 billion US$ are said to be spent for diseased children [254]. Between 1997 and 2006, also in the USA, a higher number of emergency presentations (22.7 vs. 17.4%), higher costs (>500 US$ per year: 55.8 vs. 45%), and an increased number of medical consultations (33.6 vs. 22.3%) were reported. Indirect costs of RS were caused by the loss of 5.67 working days in comparison to 3.74 working days (without sinusitis, calculated for 12 months each). This loss of productivity corresponds to the percentages of bronchial asthma (5.79 working days per 12 months) [255].

### 6.2 Data on CRS

In the USA in 1994, the direct costs of CRS treatment were estimated to 4.3 billion US$ [256], in 1996 to 4.5 billion US$ [254]. Despite these enormous sums, the authors already mentioned at that time that the costs associated with sinusitis were rather underestimated [254].

In the USA, based on the encoding as CRS (ICD-9-CM 473) representative surveys could reveal a total of 14,419,000 medical consultations in 2005. About 75% were made by general practitioners, 8.3% were made by surgically working physicians, 4.4% by conservatively working specialists, and further 8.4% sought medical assistance in outpatient departments of hospitals. Additionally 4.1% presented as emergency cases in hospitals [257].

The total number of outpatient medical contacts because of CRS is stable in comparison to 1997 (14,907,000) [258] and also based on the annual surveys (Table 10 (Tab. 10)). In the age group of 25–44 year-old people, there were 4,450,000±442,000 outpatient presentations in 2001/2002 which corresponds to 1.8±0.2% of all outpatient contacts in this age group and makes chronic sinusitis the ninth frequent diagnosis for these ages. Thus CRS causes four times as many outpatient treatments as acute sinusitis (3,654,000) [259].

Within the discipline of otolaryngology, only presentations because of otitis media or functional disorders of the Eustachian tube occurred more often in 2001 with 13,993,000 [260].

Those data become even more descriptive when they are calculated as costs per patient.

In a prospective investigation, the direct costs of 921 US$ per patient per year were calculated. They were caused per year by 2.7 antibiotic treatments and the application of nasal steroids over 18.3 weeks or antihistamines over 16.3 weeks [261].

From an economic point of view the outpatient control examinations primarily served for follow-up prescriptions of drugs (7.89 vs. 5.52 prescriptions) [256]. In this context, increasingly contacts of general practitioners (sinusitis: 5.58 contacts of general practitioners, 2.31 contacts of specialists; all patients: 3.51 contacts of general practitioners, 2.01 contacts of specialists) were observed. A differentiation of the role that ENT specialists play was not included in the investigation.

The medical treatment costs were consecutively increased in the group of CRS patients by 28% (US$ 354.06 vs. US$ 275.72). 59.7% of those costs were caused by antibiotics, 20.9% by (topical) steroids, and 19.4% by decongestants and antihistamines.

Additionally, indirect costs are due to 73 million days of reduced activity per year (in 1992 [262]. Prospectively, the follow-up of 322 CRS patients revealed a loss of 4.8 working days per 12 months [261]. Based on US American data, Bhattacharyya reported a CRS associated work loss per patient of 1.04±0.39 days. Based on a CRS prevalence of 4.9±0.2% [263] he calculated the indirect costs of CRS amounting to 11.5 million days of work loss [264].

Current data from a population-based investigation from Taiwan confirm a higher number of outpatient medical consultations for CRS patients (3.9 vs. 1.4) with higher costs (US$ 77.7 vs. US$ 19.4) [251].

Thus CRS causes relevant direct and indirect costs in the health care system. Because of the age of the presented data, the confirmed prevalence of CRS of 10.9%, and the mentioned knowledge regarding new comorbidities of CRS, the economic potential of CRS in Germany is probably even higher than described here.

### 6.3 Economic data on pediatric CRS

Economic data on CRS in children are rarely described because sinusitis is evaluated generally together with allergic symptoms.

For children below the age of 15, there are data for 2001/2002 according to which children with chronic sinusitis are responsible for 1.8±0.2% of all outpatient medical consultations (3,418,000±416,000) [259]. So 5.6±0.7 presentations of 100 children were made because of CRS. Accordingly, chronic sinusitis is the eighth most common diagnosis in children. Considering the age group of 15–24 year-old patients CRS ranges in sixth place of the reasons for medical presentations (2,054,000±282,000; 2.1±0.3% of all presentations of this age group) [259].

### 6.4 Economic parameters from Germany

**Figure 2:**
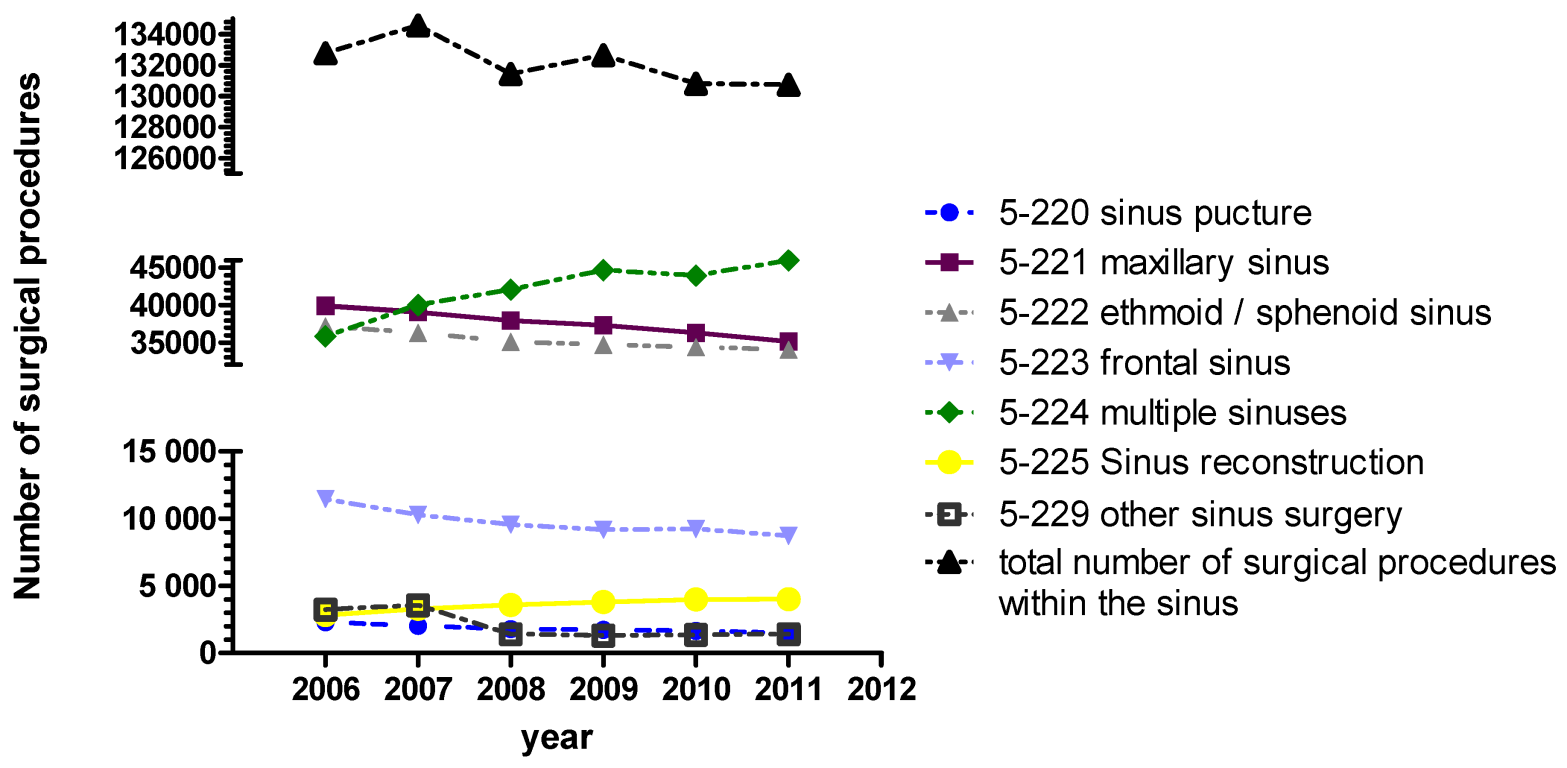
Number of sinus surgeries in Germany (2006–2011) This figure shows the number of performed procedures according to OPS code and their total number classified per year in Germany according to the health care reports of government (www.gbe-bund.de).

**Figure 3:**
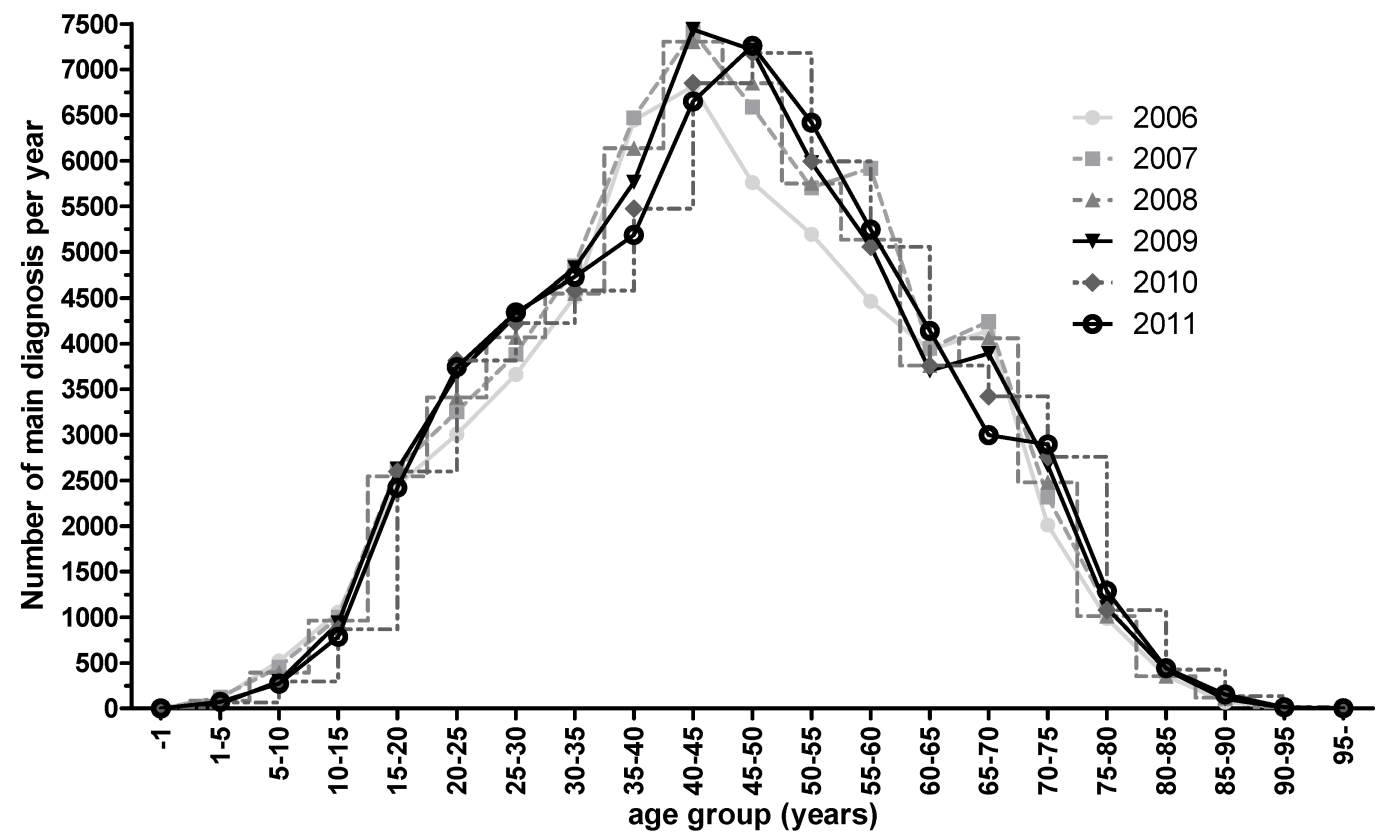
Number of hospital main diagnoses “CRS” in Germany (2006–2011) The figure shows the number of main diagnoses according to the ICD code per year and age group of the patients according to the health care reports of government (www.gbe-bund.de).

Currently according detailed data are missing for Germany. However, health reports of the federal government allow extraction of the following data regarding paranasal sinus interventions: Basically the total number of sinus surgeries was relatively constant between 2006 and 2011 (mean value ± standard deviation: 132,165±1,474; Figure 2 (Fig. 2)). Also the age structure of the patients undergoing surgery was similar in the observation period (Figure 3 (Fig. 3)).

While “other interventions” slightly decrease, the number of interventions of several paranasal sinuses (OPS 5-224) has increased. In this context it must be mentioned that DIMDI (http://www.dimdi.de) has added the codes 5-222.8 “balloon dilatation of the sphenoid sinus entrance”, 5-223.7 “balloon dilatation of frontal sinus entrance”, and 5-224.8 “balloon dilatation of the entrances of several paranasal sinuses” since 2010 and removed the subparagraphs of the OPS codes 5-229 since 2008. Those changes cannot be quantified based on the data at our disposition. Thus it cannot be stated that in Germany the introduction of balloon dilatation has led to more generous indications for surgery.

According to the data originating from the German Law of Hospital Fees (§21 KHEntgG) of 2009 (data available from May 31, 2010), a detailed analysis was performed with support of the Institute for Hospital Fee Systems (InEK).

The diagnoses and procedures are limited to the 20 most common ones because of the data processing of the InEK. The diagnoses are restricted to the field of chronic sinusitis or polyps of the paranasal sinuses (J32.0–J32.9 and J33.8, without mucoceles or cysts). It must be mentioned for the calculated percentages that they are set in relation to the total number of the 20 most common diagnoses or procedures (TOP20) and not to the total number of interventions. Regarding the secondary diagnoses, multiple answers were possible. In the presented tables the number of the cases are listed and not the number of answers in order to estimate the incidences conservatively.

In the field of day-care patients, 10/96 main diagnoses (10.4% TOP20; DRG D06A: 0 cases, D06B: 0 cases; D06C: 10 cases) were made in the field of CRS. As secondary diagnoses, CRS was reported in 7/100 cases (7%). Further, 18/256 procedures (Dp6A: 0; D06B: 2; D06C: 16 procedures) or 7% of the TOP20 were performed in the area of the paranasal sinuses.

Regarding the specialized ENT units 17,280/28,027 main diagnoses (50.5% TOP 20; DRG D06A: 0 cases, D06B: 266 cases; D06C: 17,018 cases) were made in the context of CRS. Those departments observed 30,675/72,708 (D06A: 6; D06B: 217; D06C 30,452) procedures (Table 11 (Tab. 11)) or 41.6% of the TOP20 procedures in the area of the paranasal sinuses. Thus, sinus surgeries are performed in 44% (D06A: 7%; D06B: 8%; D06C: 45.4%) of the surgical TOP20 procedures of German ENT departments for the use of practicing physicians in 2009.

Specialized ENT Hospitals reported 31,877/57,158 (55.8% TOP20; DRG D06A: 17 cases; D06B: 563 cases; D06C: 31,297 cases) main diagnoses from the field of CRS. As secondary diagnoses, CRS was coded in 50,459/97,730 cases (51.6% TOP20; DRG D06A: 0; D06B: 255; D06C: 50,204). All procedures of the paranasal sinuses performed in main wards were coded as DRG D06C procedures. A total of 55,858 procedures were performed at the paranasal sinuses, corresponding to 31.4% of the total of coded TOP20 procedures. Concentrating on surgical procedures, the interventions at the paranasal sinuses represented 48.7% of the surgical TOP20 (partial) interventions of those 3 DRGs in 2009 (Table 12 (Tab. 12)).

These data explain that the activity of outpatient physicians as well as main wards contribute significantly to the volume of CRS treatments according to DRG D06A, D06B, and D06C. So CRS and its treatment play a relevant role in the continuous existence of the ENT discipline and regarding the reputation in the population. In summary with the American data on the direct and indirect costs of CRS, a significant relevance becomes obvious also from an economic point of view.

## 7 Conclusion

Because of the high incidence of CRS in the population as well as the interaction with other common diseases such as bronchial asthma, acute myocardial infarction, and apoplexy but also depression, an extensive epidemiologic investigation on the incidence of CRS and its subtypes, the influence of comorbidities, and the direct and indirect costs caused by CRS would be desirable from an ENT specific point of view. The presented data show a high medical and health care economic relevance of CRS in Germany and simultaneously they indicate the poor and unsatisfactory study situation. Furthermore it is not recommended to uncritically adopt data from other regions of the world because of several differences (of the population, but also the health care systems).

The European investigation reveals a clear prevalence for the region of Duisburg with 14.1% and for Brandenburg with 6.9%. In Duisburg (8.4%) as well as in Brandenburg (4.6%), the physician-based prevalence was definitely lower than a symptom-based diagnosis based on the EPOS3 criteria. So there seems to be a clear regional variation within Germany that presents an enormous chance to retrieve data about the still unknown pathophysiology of CRS. Beside environmental influences (urban regions reveal a rather high CRS prevalence in Europe), another reason might be a difficult access to ENT specialists in rural regions such as Brandenburg. Also the different status of industrial development, nutrition, and life circumstances during the German Division must be mentioned as possible influence factors.

With this background it is unfortunate that the large representative investigation in Germany of this year, the so-called “National Cohort” (http://nationale-kohorte.de/) will not deal with chronic rhinosinusitis despite the objective to research on the origins of common diseases and to define risk factors. However, the medical consultation of specialists, smoking, and bronchial asthma will be part of the investigation so that in the context of planned subsequent investigations hopefully aspects of pathophysiology and pathogenesis of chronic rhinosinusitis may be clarified.

In spite of the heterogeneity of the described data, a relevant prevalence of CRS could be confirmed for all continents. We hope that those data will attract the attention of patients, ENT specialists, and decision making authorities in order to fill the mentioned gaps in knowledge.

## Notes

### Competing interests

The author declares that he has no competing interests.

## Figures and Tables

**Table 1:**
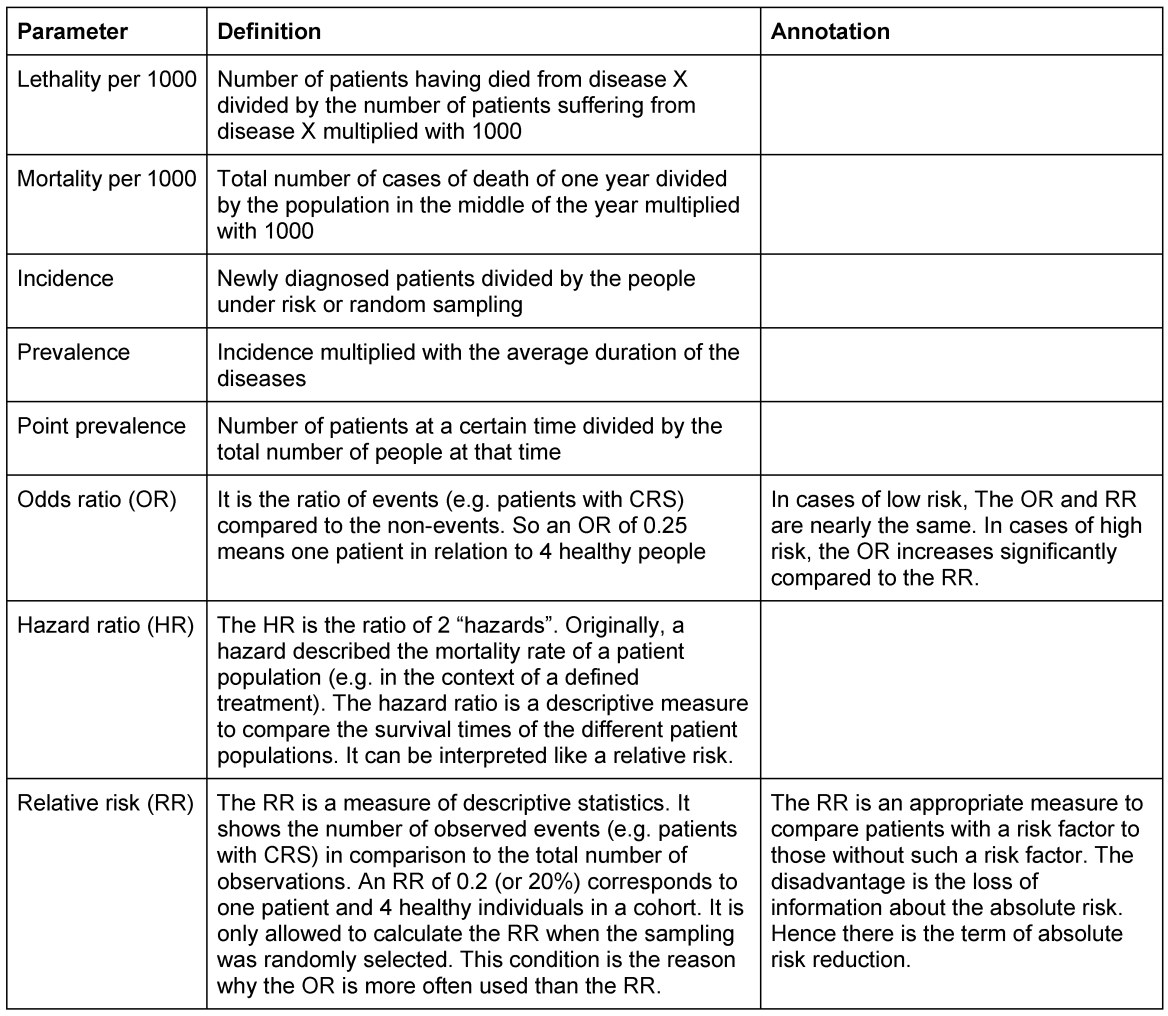
Epidemiologic parameters and their definition The table gives the typical epidemiologic terms, their definition, and if appropriate annotations for better differentiation and understanding.

**Table 2:**
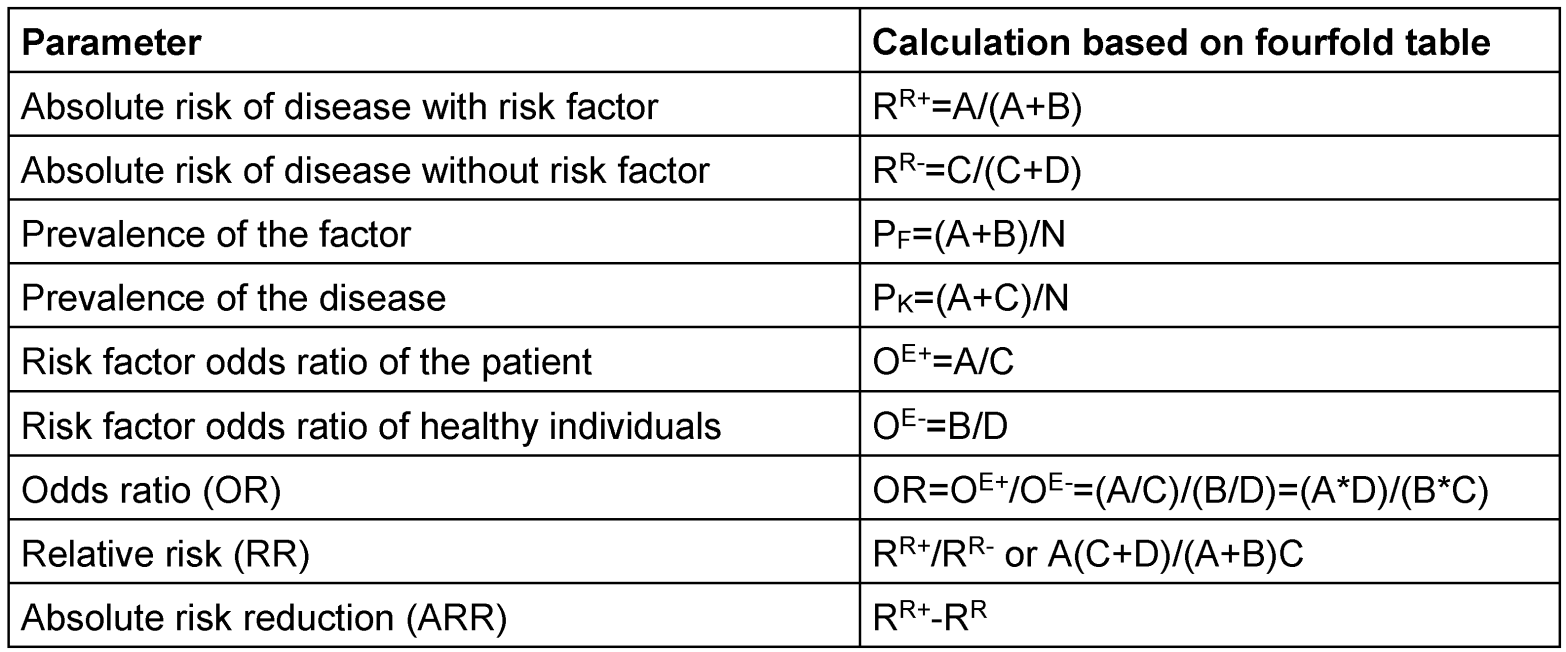
Calculation of epidemiologic parameters The table gives epidemiologic parameters and their calculation formula based on a fourfold table.

**Table 3:**
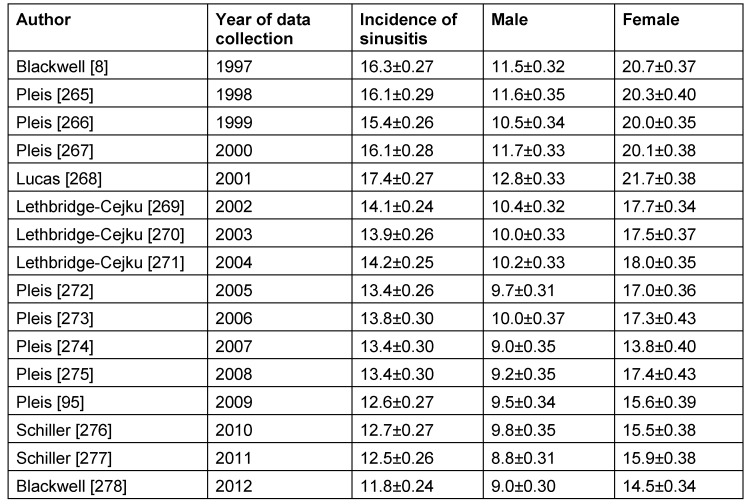
Result of representative surveys carried out among the adult population of the USA regarding the incidence of rhinosinusitis The table shows the results from the USA over the years regarding the anamnestic incidence of sinusitis in the total population and classified according to the gender.

**Table 4:**
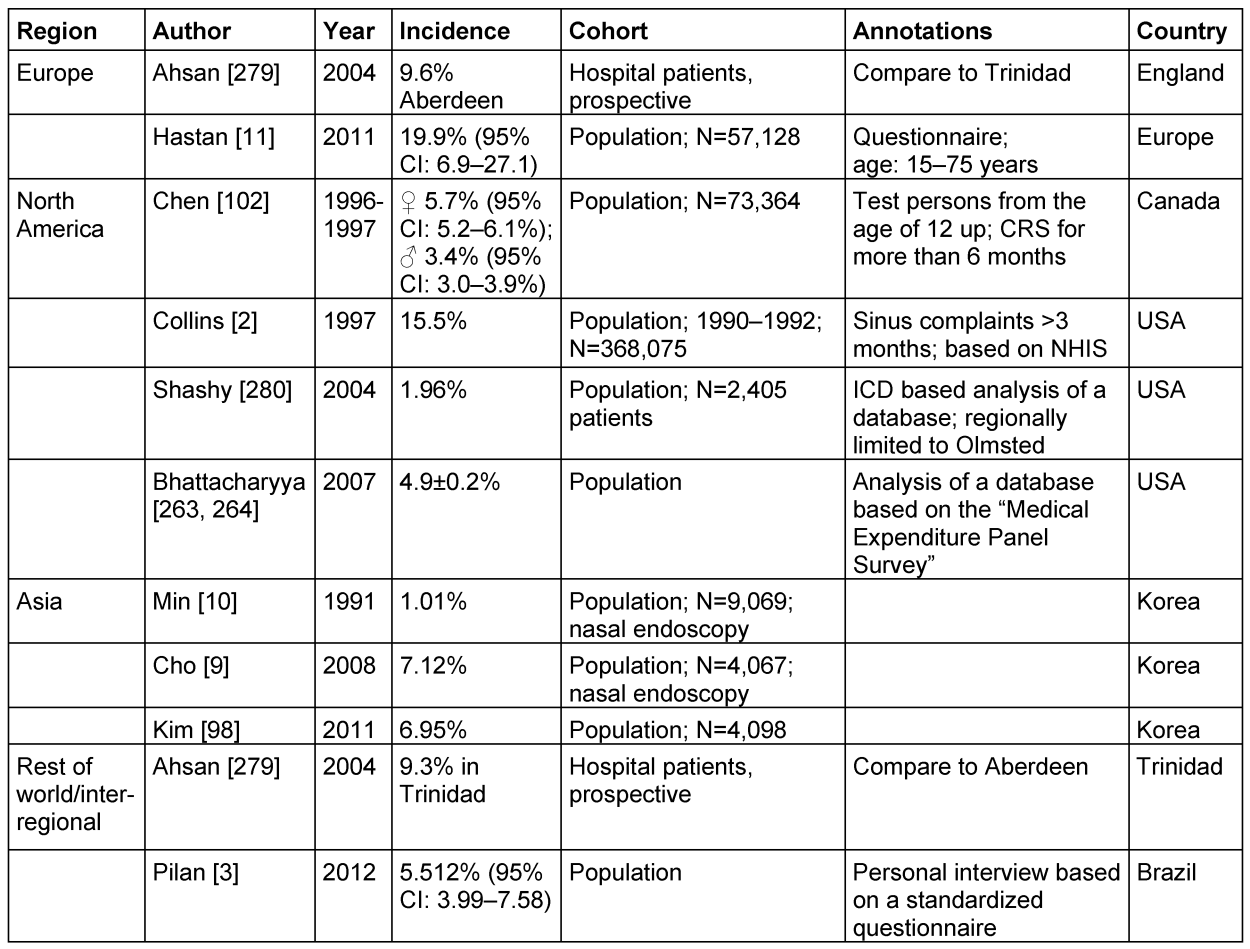
Epidemiologic data on CRS The table shows the region where the investigation took place (“region”), the first author of the paper (“author”), the year of data collection or if not mentioned otherwise of the publication (“year”), the incidence of CRS (if possible with confidence interval), annotations on the methods as well as the country where the investigation had been performed.

**Table 5:**
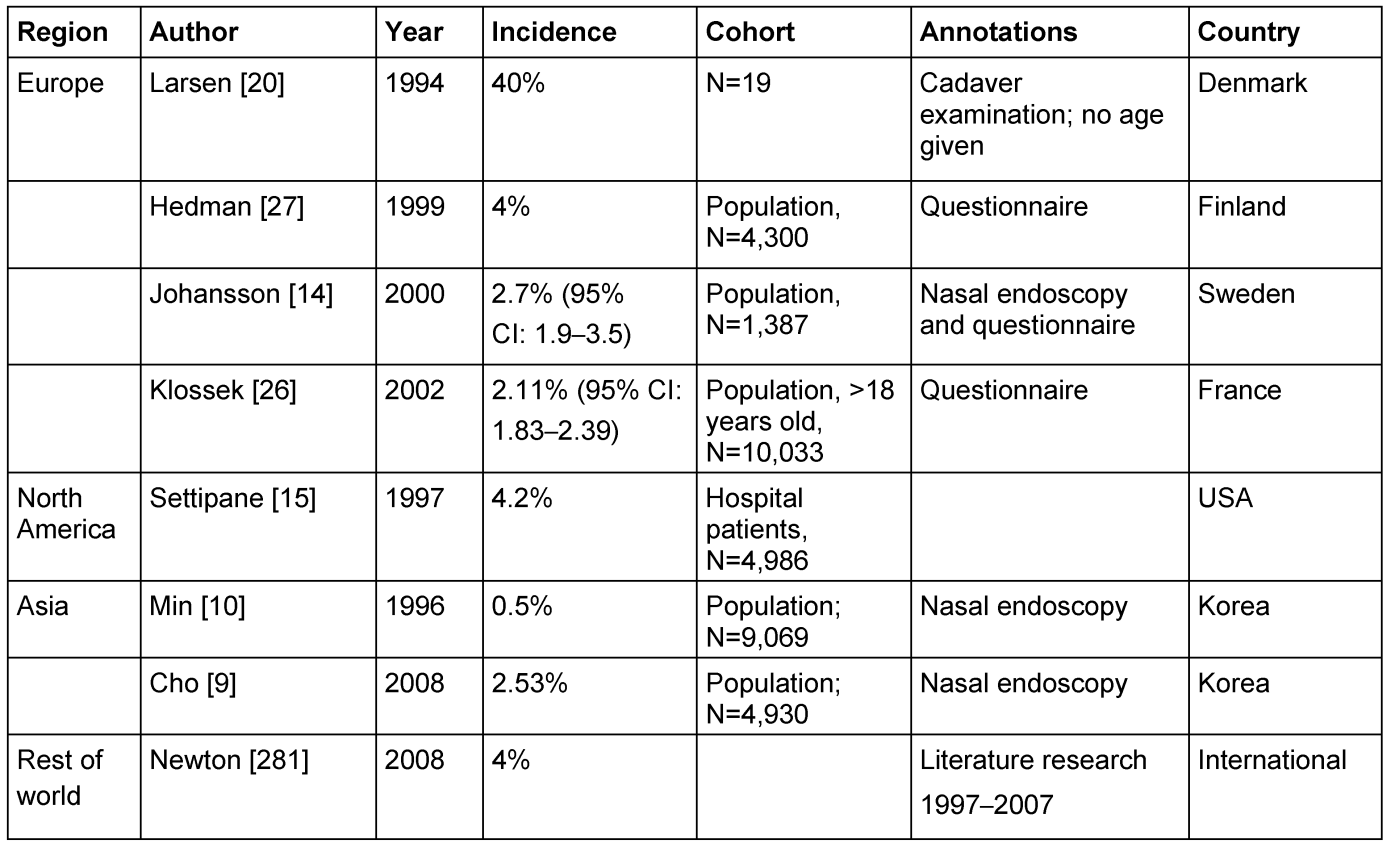
Current epidemiologic data on CRSwNP The table shows the region where the investigation took place (“region”), the first author of the paper (“paper”), the year of data collection or if not mentioned otherwise of the publication (“year”), the incidence (if possible with confidence interval), annotations on the methods as well as the country where the investigation had been performed. The data of this table refer to the paragraph of “Special subtypes of CRS, CRS with and without identification of nasal polyps”.

**Table 6:**
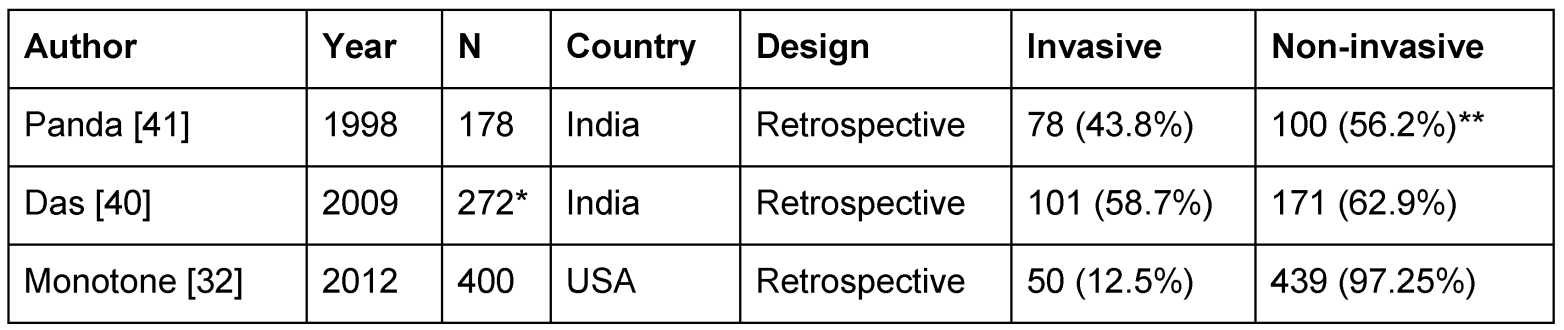
Incidence of invasive and non-invasive funcal sinusitis The table shows the authors, the number of included patients, the year, and the country of data collection or publication also the method of the studies as well as the given absolute and relative (%) number of patients according to the diagnosis. *=study without mixed form; **=8 patients with AFS were added to the non-invasive group for better comparability.

**Table 7:**
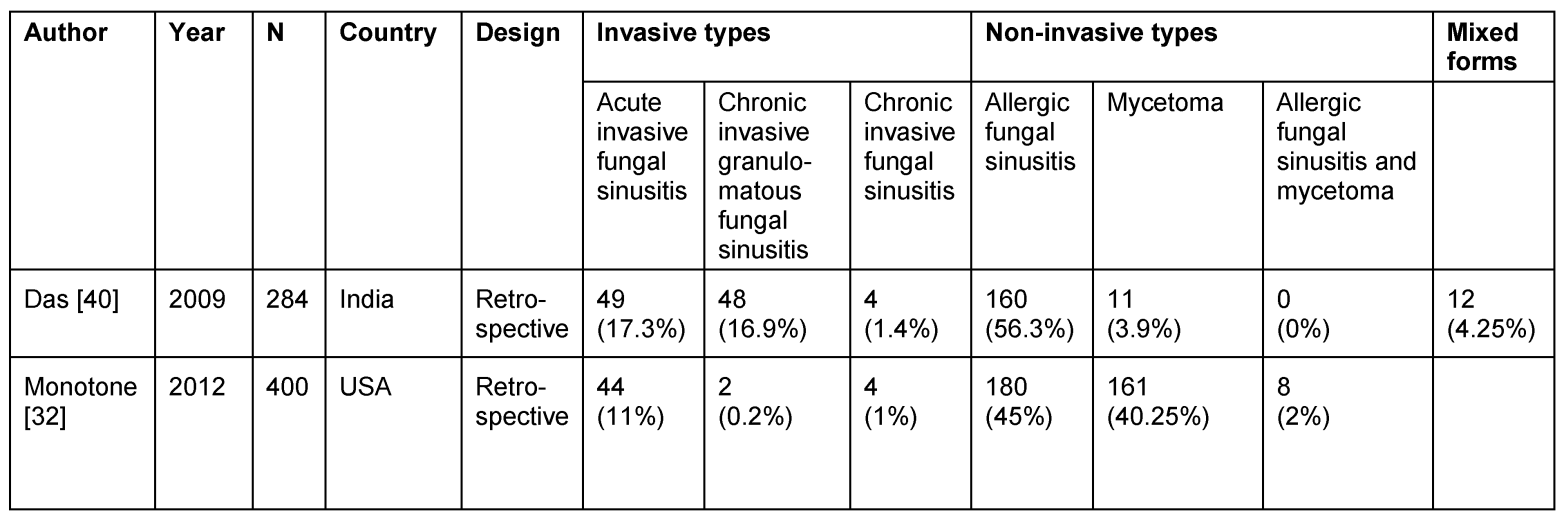
Subtypes of fungal sinusitis and their incidence The table shows data on the author, the included number of patients (N), the year and country where the study was performed or published and also the method of the study as well as the absolute and relative (%) number of patients according to the diagnosis. In contrast to Tab. 6 the subtypes of fungal sinusitis are considered.

**Table 8:**
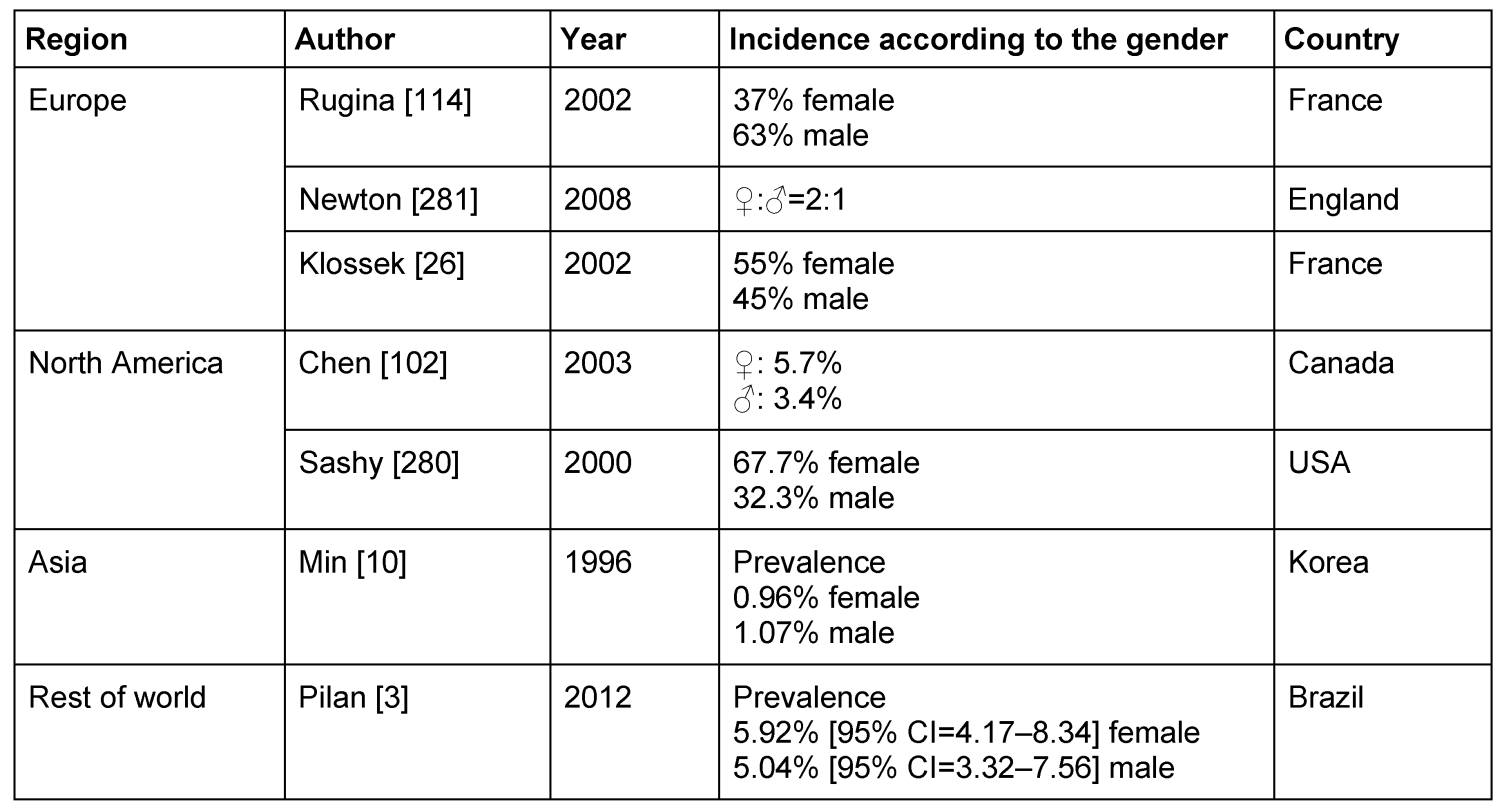
Selected investigations with data on the distribution of the gender The table shows selected studies with data on the incidence of CRS according to the gender. Because of the data quality, the incidence is given as gender-specific prevalence rate, alternatively as ratio.

**Table 9:**
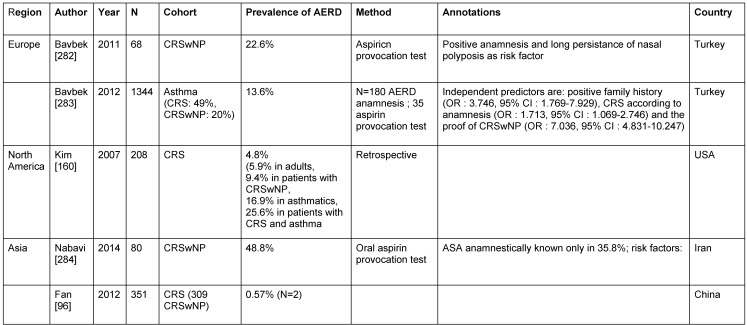
Incidence of AERD in selected patient populations The table shows data on the region, the first author (“author”), year and number of included patients (“N”), completed by the significant inclusion criterion in relation to CRS, the prevalence of AERD, the method if provocation tests were applied and observed risk factors, and the country where the investigation was performed.

**Table 10:**
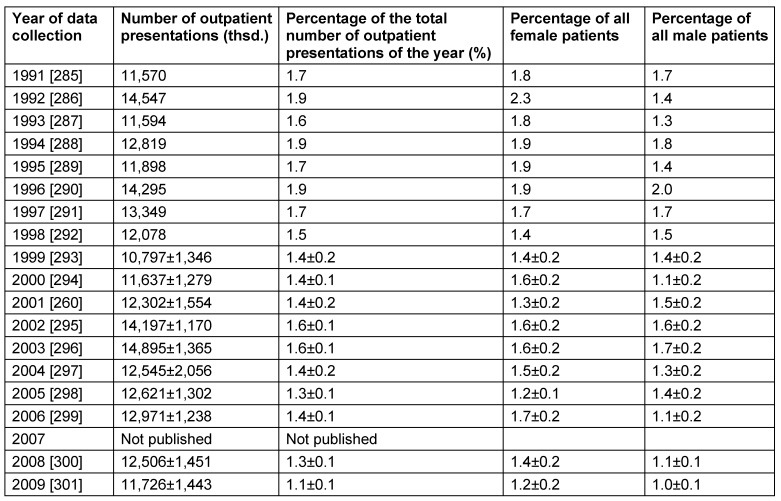
Number of outpatient presentations because of CRS in the USA according to the National Ambulatory Medical Care Survey (NAMCS) The analysis was performed based on the ICD code (code 472). The table shows the average values, the standard deviation is given if published, as well as the gender distribution of the patients.

**Table 11:**
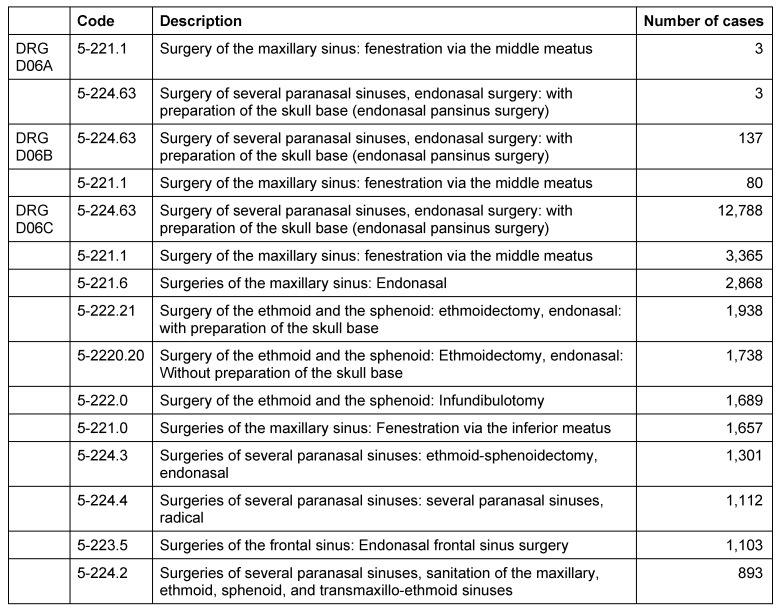
Number of the sinus surgeries (cases) performed by practicing physicians according to InEK in 2009 The cases are classified according to the DRG system. The table shows the cases performed by practicing physicians classified according to DRG with OPS code, description, and number of 2009 based on InEK.

**Table 12:**
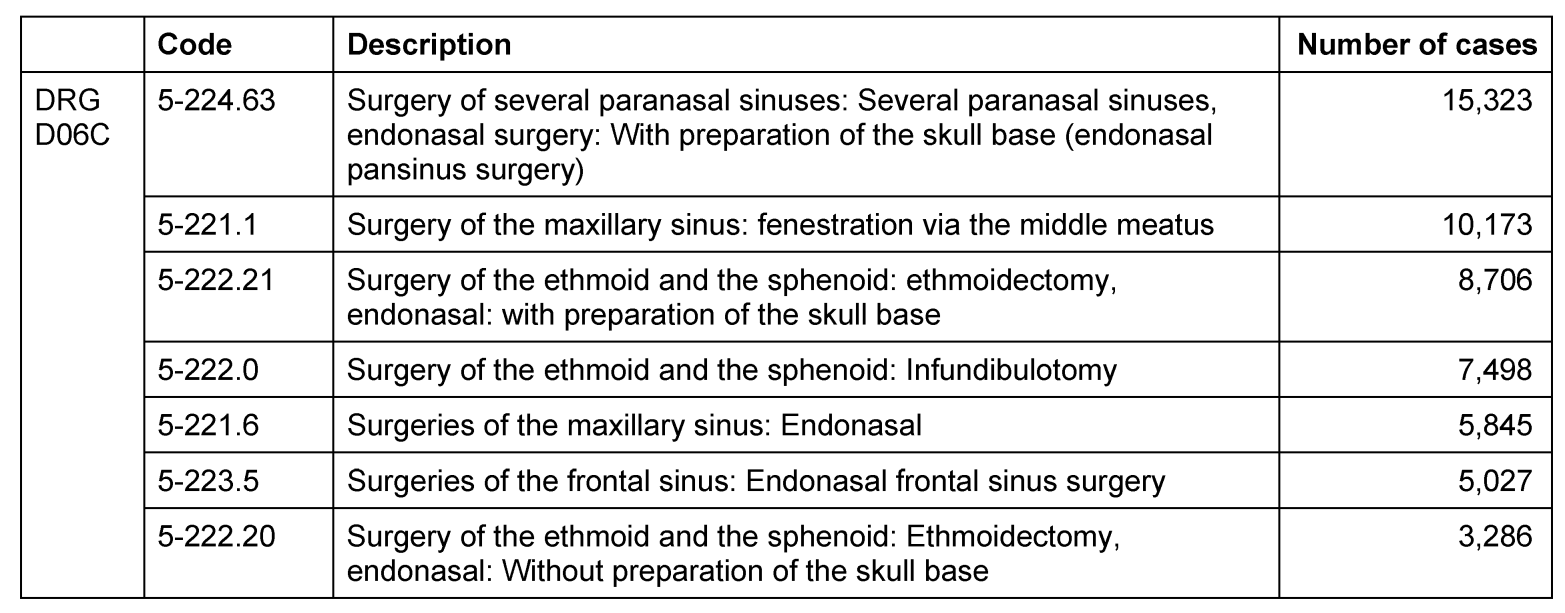
Number of paranasal sinus interventions performed by ENT Departments in 2009 according to InEK The table shows the cases that have been settled by ENT Departments in Germany, listed according to the DRG with OPS code, description, and number according to InEK in 2009.
